# Asymmetric electron acceptor enables highly luminescent organic solar cells with certified efficiency over 18%

**DOI:** 10.1038/s41467-022-30225-7

**Published:** 2022-05-11

**Authors:** Chengliang He, Zeng Chen, Tonghui Wang, Ziqiu Shen, Yaokai Li, Jiadong Zhou, Jianwei Yu, Huiyu Fang, Yuhao Li, Shuixing Li, Xinhui Lu, Wei Ma, Feng Gao, Zengqi Xie, Veaceslav Coropceanu, Haiming Zhu, Jean-Luc Bredas, Lijian Zuo, Hongzheng Chen

**Affiliations:** 1 State Key Laboratory of Silicon Materials, MOE Key Laboratory of Macromolecular Synthesis and Functionalization, Department of Polymer Science and Engineering, Zhejiang University, 310027 Hangzhou, P.R. China; 2 Department of Chemistry, Zhejiang University, 310027 Hangzhou, P.R. China; 3 Department of Chemistry and Biochemistry, The University of Arizona, Tucson, AR 85721-0088 USA; 4 Institute of Polymer Optoelectronic Materials and Devices, State Key Laboratory of Luminescent Materials and Devices, South China University of Technology, 510640 Guangzhou, P.R. China; 5 Department of Physics, Chemistry, and Biology, Linköping University, Linköping, SE-58183 Sweden; 6 State Key Laboratory for Mechanical Behavior of Materials, Xi’an Jiaotong University, 710049 Xi’an, P.R. China; 7 Department of Physics, Chinese University of Hong Kong, New Territories, 999077 Hong Kong, P. R. China; 8 Zhejiang University-Hangzhou Global Scientific and Technological Innovation Center, 310014 Hangzhou, P. R. China

**Keywords:** Devices for energy harvesting, Electronic devices, Solar cells

## Abstract

Enhancing the luminescence property without sacrificing the charge collection is one key to high-performance organic solar cells (OSCs), while limited by the severe non-radiative charge recombination. Here, we demonstrate efficient OSCs with high luminescence via the design and synthesis of an asymmetric non-fullerene acceptor, BO-5Cl. Blending BO-5Cl with the PM6 donor leads to a record-high electroluminescence external quantum efficiency of 0.1%, which results in a low non-radiative voltage loss of 0.178 eV and a power conversion efficiency (PCE) over 15%. Importantly, incorporating BO-5Cl as the third component into a widely-studied donor:acceptor (D:A) blend, PM6:BO-4Cl, allows device displaying a high certified PCE of 18.2%. Our joint experimental and theoretical studies unveil that more diverse D:A interfacial conformations formed by asymmetric acceptor induce optimized blend interfacial energetics, which contributes to the improved device performance via balancing charge generation and recombination.

## Abstract (web summary)

High-performance organic solar cells call for novel designs of acceptor molecules. Here, He et al. design and synthesize a non-fullerene acceptor with an asymmetric structure for diverse donor:acceptor interfacial conformations and report a certificated power conversion efficiency of 18.2%.

## Introduction

Organic photovoltaics (OPV) is an emerging technology that presents great potential in terms of renewable energy source, due to specific advantages of organic materials, such as flexibility, low cost, selective absorption for visible transparency, vivid colors, etc. With comprehensive efforts in molecular design, morphology control, interface engineering, and device architecture, the current record power conversion efficiency (PCE) has reached 19%, which pushes OPV to the edge of commercialization^1–8^. However, the PCEs remain lower than those of inorganic materials, e.g. silicon (PCE up to 26.7%) or GaAs (29.1%)^5^, as a result mainly of the larger open-circuit voltage (*V*_oc_) loss that penalizes organic solar cells (OSCs) (*V*_oc_ loss ~0.55 eV for the state-of-the-art PM6:Y6 systems)^4,9–11^.

The recent development of non-fullerene acceptors (NFAs) has produced charge-transfer (CT) state energies close to that of the strongly absorbing (and emitting) local-exciton (LE) state on the donor and/or acceptor, which has contributed to significantly reduced *V*_oc_ loss^11,12^. This is a major advance compared to fullerene-based OSCs, whose large *V*_oc_ loss is typically related to a much lower CT-state energy compared to the LE-state energy^13–15^. In addition, non-radiative decay can be significantly reduced in NFA-based devices^16,17^. Overall, the best performance from first-generation NFAs, e.g., IT-4F or its derivatives, reaches over 15% PCE^18^, while the emergence of Y6 and its derivatives has brought PCEs over 18%. Y6 has a banana shape and a rather porous three-dimensional (3D) packing pattern, which results in remarkable optoelectronic properties, including a low exciton-binding energy and ambipolar charge transport ability with high carrier mobilities^8,10,19,20^.

When considering the evolution of the acceptor molecular symmetries (from the fullerene family to the ITIC family and the Y6 family), it is most interesting to realize that the degree of molecular symmetry gradually reduces, from the ball-shaped (icosahedral) fullerenes, to ITIC molecules with a largely coplanar core and inversion symmetry, to Y6 molecules with a banana-shape and a helical chiral structure. In this context, the logical next step is the development of asymmetric acceptors, a strategy that is getting increasing attention, as reported by Li et al. ^21^.

Efforts to design and synthesize asymmetric acceptors via tuning of the side chains, central cores, and terminal groups have resulted in high device performance over 18%^8,22–26^. We summarize the recently reported Y-series asymmetric NFAs in three categories: (i) Y-series NFAs with asymmetric backbones^24^; (ii) Y-series NFAs with asymmetric side chains^22,23^; (iii) Y-series NFAs with asymmetric terminals^26–28^ (see chemical structures in Supplementary Fig. 1 and device parameters in Supplementary Table 1). The molecular symmetry is expected to affect directly the molecular packing and optoelectronic properties; asymmetric molecules typically exhibit larger dipole moments and stronger intermolecular interactions, which could lead to more efficient intermolecular packing. However, we are still lacking in fundamental understandings of the specific characteristics brought by asymmetric molecules in terms of molecular conformations, energetics, and optoelectronic properties, which is highly desirable to facilitate the development of the next generation high-performance OSCs. In this work, we present a deep understanding on how asymmetric NFAs with indanone and cyano indanone terminals affect the device performances from multiple points of view of observation of molecular packing in solid state, theoretical calculation for intermolecular interaction, charge dynamic behaviors, etc.

**Fig. 1:**
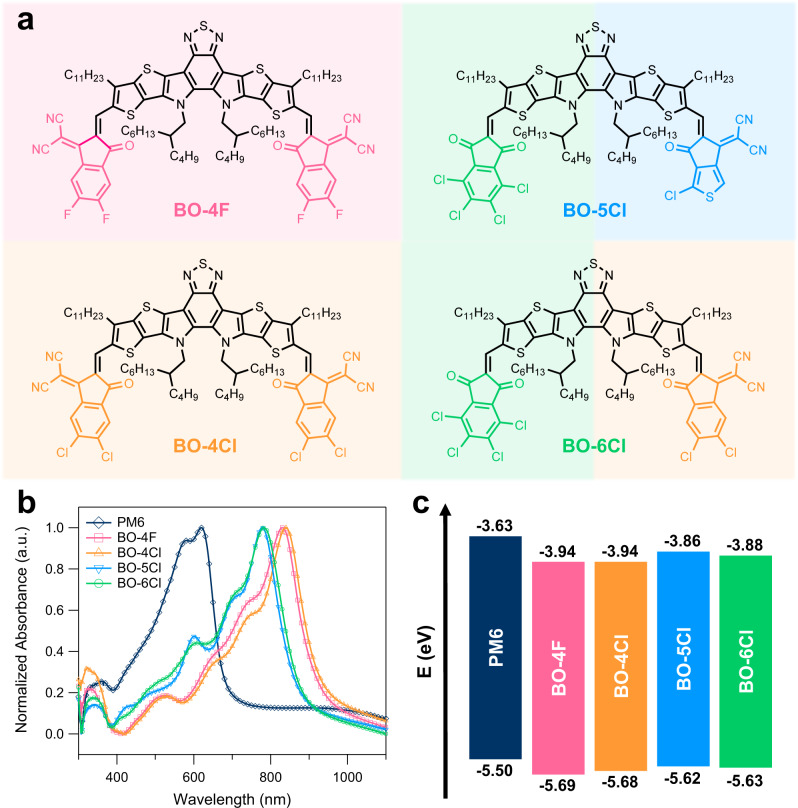
Basic characteristics of the target acceptors. **a** Chemical structures of the four acceptors. **b** UV–vis absorption spectra of thin films. **c** Energy-state diagram determined by cyclic voltammetry.

Here, based on the design of the BO-5Cl molecule (see Fig. 1) that displays different electron-poor terminals (indanone and cyano indanone terminals), we report that such asymmetric acceptors can lead to OSCs that combine low non-radiative voltage losses and high charge generation efficiency, due to the dual nature of the interfacial electronic states formed by more diverse molecular conformations in donor:acceptor (D:A) interfaces. The bulk-heterojunction (BHJ) active layer based on BO-5Cl and the representative polymer donor PM6 displays an high electroluminescence external quantum efficiency (EQE_EL_) of 10^−3^ and EQE_PV_ approaching 80%. Further, adding an asymmetric molecule as the third component will induce similar effect as that in binary blend, i.e. the more diverse D:A interfacial conformations over the symmetric ones. As a result, the impact of exploiting the asymmetric BO-5Cl molecule as third component also manifests in ternary blends with PM6 and BO-4Cl, as it leads to high-performance devices with a PCE of 18.56% (certified as 18.2%), which is among the highest certified PCE values.

## Results and discussion

### Synthesis and characterization

The asymmetric molecular structures of BO-5Cl and BO-6Cl are shown in Fig. 1a. For the sake of comparison, the symmetric BO-4F and BO-4Cl NFAs have also been synthesized (Fig. 1a). BO-4F is similar to the commonly used Y6 but has longer side chains on the central core. BO-4Cl is obtained by replacing the F atoms on BO-4F with Cl atoms. BO-6Cl has been reported in our previous work as BTP-S2^27^. These molecules represent the current state-of-the-art in terms of performance of Y-series NFAs^8,27,29,30^. The synthetic routes of the four acceptors are illustrated in Supplementary Fig. 2. These acceptors are developed by conjugating the end-groups to the BTP (12,13-dihydro-[1,2,5]thiadiazolo[3,4-e]thieno[2″,3″:4′5′]thieno[2′,3′:4,5]pyrrolo [3,2-g]thieno[2′,3′:4,5]thieno[3,2-b]indole) core through Knoevenagel condensation. 5,6-Difluoro-3-(dicyanomethylene)indanone (IC-2F) and 5,6-dichloro-3-(dicyanomethylene)indanone (IC-2Cl) are utilized to synthesize BO-4F and BO-4Cl, respectively. To minimize energy-state offsets, 4,5,6,7-tetrachloroindane-1,3-dione (IO-4Cl) is introduced to replace one side of BO-4Cl, leading to the BO-6Cl molecule^27^. By adopting 2-(1-chloro-6-oxo-5,6-dihydro-4H-cyclopenta[c]thiophen-4-ylidene) malononitrile (T-Cl) on one side while maintaining IO-4Cl on the other side, we obtain the BO-5Cl molecule that has a lower ionization potential (crudely speaking, an upshifted highest occupied molecular orbital (HOMO) level). The detailed synthesis protocols and structure characterizations can be found in Supplementary Figs. 3–7. All the acceptors exhibit good solubility in common solvents such as chloroform and chlorobenzene.

The optical properties of the four acceptors were characterized by UV–vis absorption and fluorescence spectroscopies (Fig. 1b and Supplementary Fig. 8). The maximum absorption peaks of the four acceptors in solution appear in the range of 710–740 nm (1.68–1.75 eV). The results from excited-state calculations performed at the time-dependent density functional theory (TD-DFT) level (Supplementary Fig. 19) are in line with the experimental data, suggesting a moderate (~80 meV) blue shift in absorption for BO-5Cl when compared to BO-4Cl. The natural transition orbitals (NTOs) calculated for BO-4Cl indicate that the first excited state (S_1_) has a significant intra-molecular CT character, with the hole localized on the BTP core and the electron delocalized on both end groups. A similar intra-molecular CT description is obtained for BO-5Cl, however, with the major difference that only the T-Cl end group is involved in the excitation; the S1 state of BO-5Cl is characterized by a very large state dipole moment of about 15 D, to be compared to 4 D calculated for S_1_ in BO-4Cl (Supplementary Fig. 19).

There occurs an obvious red-shift in the absorption and photoluminescence (PL) spectra when casting these NFAs into thin films (Fig. 1b and Supplementary Fig. 8). For instance, the PL peak of BO-4Cl in toluene appears at 770 nm (1.61 eV) and red-shifts to 889 nm (1.39 eV) in the film. A similar trend was also observed for Y6, as attributed to aggregation and increased exciton delocalization^9^.

Cyclic voltammetry (CV) was carried out to measure the state energies; the measured ionization potentials and electron affinities are displayed in Fig. 1c and Supplementary Fig. 9. The ionization potentials decrease from BO-4F to BO-4Cl, BO-6Cl, and BO-5Cl. Ultraviolet photoelectron spectroscopy (UPS) was conducted to cross-check the CV results (Supplementary Fig. 9). The ionization potential differences between PM6 and the NFAs are measured to be 0.076, 0.070, 0.035, and 0.026 eV for BO-4F, BO-4Cl, BO-6Cl, and BO-5Cl, respectively, which is in line with the CV results (Supplementary Tables 2–5).

### Molecular packing in single crystals and thin films

**Fig. 2:**
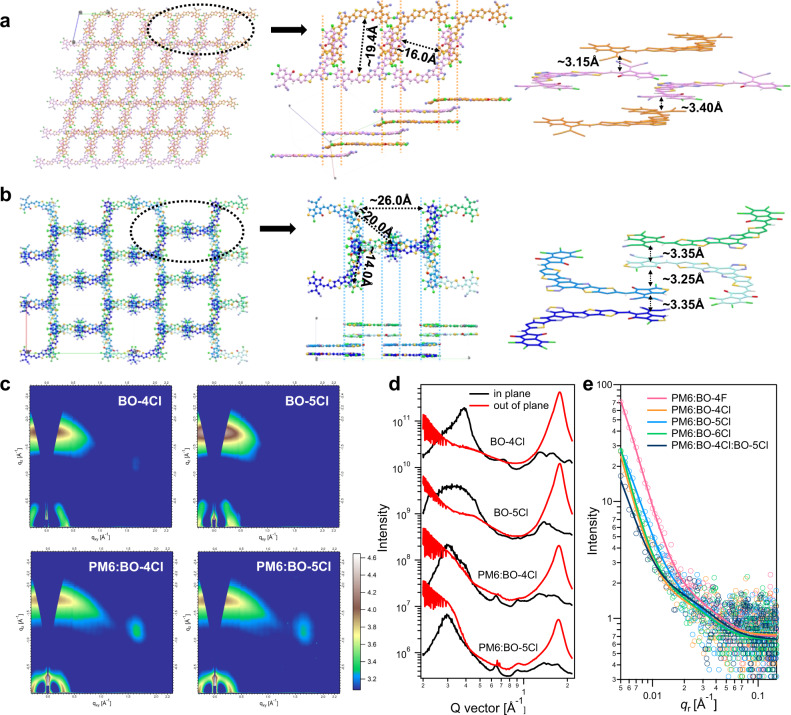
Molecular packing in single crystals and thin films. Molecular stacking patterns of **a** BO-4Cl and **b** BO-5Cl in the single crystals. **c** 2D GIWAXS images. **d** GIWAXS intensity profiles of the corresponding films along the in-plane (black lines) and out-of-plane (red lines) directions. **e** GISAXS intensity profiles of the corresponding films along the *qr* axis.

In addition to their molecular structures, the packing behavior of the NFAs has also been demonstrated to play a key role in determining their optoelectronic properties and OSC performance^31^. We first analyzed the molecular packing characteristics in single crystals of BO-4F, BO-4Cl, and BO-5Cl. Details regarding single-crystal growth can be found in the Supplementary Fig. 10. Overall, these molecules form ordered structures with 3D packing character. However, as shown in Fig. 2a, different end-groups lead to different molecular stacking patterns. For all of these NFAs, the distance between the S atom on the external ring of the core and the O atom on end-group is in the range of 2.60–2.70 Å (Supplementary Fig. 10). This O…S noncovalent interaction leads to good planarity, which is conducive to form efficient transport channels among adjacent molecules. The single-crystal stacking patterns in Y6 and BO-4F (Supplementary Fig. 11) are rather similar, with π–π stacking present not only between overlapping terminal groups (which is also found in the single crystals of the ITIC family^31^) but also between overlapping cores (as found in Y6)^32^. The numerical values highlighted in Supplementary Fig. 11b represent the shortest distances for the main charge transport channel formed by the π–π stacking, which promotes a 3D charge transport network. For BO-4Cl, similar trends are found, as shown in Supplementary Fig. 10. However, after replacing the F atoms with Cl atoms, the crystal lattice parameters change substantially. Given that the sizes of the molecules are nearly identical (Supplementary Fig. 10), the observation that more molecules are stacked in the same volume as shown in Fig. 2a indicates that the stacking in BO-4Cl becomes significantly denser. In the case of BO-5Cl (Fig. 2b), the stacking network is similar to that in BO-4F, with the distance between adjacent molecules decreasing slightly from 3.4 to 3.3 Å, and the distance between stacking columns (blue dotted line) decreasing from 30.0 to 26.0 Å (Fig. 2b and Supplementary Fig. 11).

The electronic-structure calculations indicate that the transfer integrals (electronic couplings) related to electron transport are significant (larger than 50 meV) in both BO-4Cl and BO-5Cl crystals (Supplementary Fig. 20). The brick-wall structural arrangement (Supplementary Fig. 21) allows for efficient in-plane electron transport. In the BO-5Cl crystal, this in-plane electron transport takes place via two alternating hopping channels characterized by transfer integrals of 44.6 and 37.7 meV. Two additional transfer integrals of about 59.6 and 11.4 meV contribute to two different (out-of-plane) electron transport pathways along the molecular stacks^33^. A similar picture is obtained for BO-4Cl (Supplementary Fig. 21). Overall, these electronic-structure results demonstrate that the packing motifs in BO-4Cl and BO-5Cl support the existence of 3D electron transport networks in both crystals.

Grazing-incidence wide-angle and small-angle X-ray scattering (GIWAXS/GISAXS) characterizations were performed to investigate the morphology of the pristine acceptor films (Fig. 2c–e, Supplementary Figs. 12 and 13) and their blend films^34^. For the pure acceptor films, strong peaks at *q*_*z*_ = 1.80 Å^−1^ (*d* = 3.50 Å) in the out-of-plane direction and at *q*_*xy*_ = 0.390 Å^−1^ (*d* = 16.0 Å) in the in-plane direction are observed, which indicates a dominant face-on orientation of the molecular stacking (detailed packing parameters are collected in Supplementary Tables 13 and 14). The π–π stacking distances in spin-coated films are consistent with the single-crystal results. In addition, the lamellar stacking distances are close to the distances highlighted in Fig. 2a, b and Supplementary Fig. 11, that is, the molecular packing in annealed films resembles the packing motif observed in the single crystals. The GIWAXS results for the blend films are shown in Fig. 2c, d. Among them, BO-5Cl possess the highest coherence length values of the lamellar packing in the IP direction both in pristine and blend films, indicating that BO-5Cl is less amorphous than others, which may be ascribe to its large dipole moment for enhanced intermolecular interaction. Interestingly, the data suggest that the packing motifs observed in the single crystals of the acceptor molecules are retained in the PM6:A blends. Taking BO-4Cl as an example, the peaks located at *q*_*z*_ = 1.78 Å^−1^ (*d* = 3.53 Å) and *q*_*r*_ = 0.389 Å^−1^ (*d* = 16.2 Å) in the single crystal are preserved in the PM6:BO-4Cl blend film. It can be anticipated that the molecular packing in the pure acceptor domains of the blend film is similar to the single-crystal stacking pattern^9^. This means that the transport networks discussed above for the acceptor films are also expected to be inherited into the D:A blends^9^.

Figure 2e shows the GISAXS intensity profiles. Here, the scattering contribution from the pure acceptor phase is fitted by a fractal-like network model. The Debye–Anderson–Brumberger (DAB) model is used to quantify the size of amorphous intermixing region^35^. The acceptor domain sizes (2*R*_g_) of the five blends are similar (9–12 nm), all within the appropriate range for efficient exciton dissociation; the intermixing domain size (*ξ*) of PM6:BO-5Cl is the smallest, which will facilitate the charge collection from this region (the data are summarized in Supplementary Table 15).

### Excited-state dynamics

**Fig. 3:**
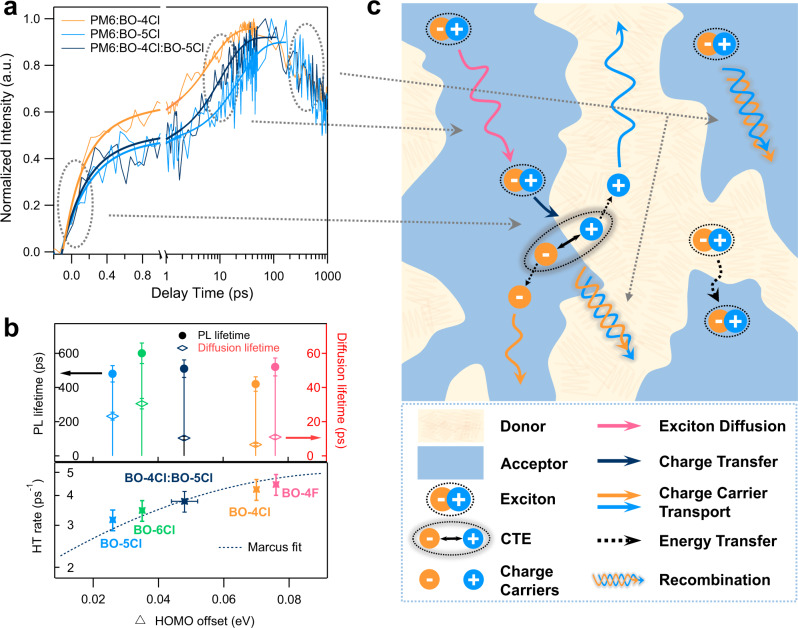
Excited-state kinetics unveiled by transient absorption spectroscopy. **a** TA dynamic curves of the three blends. **b** Relation graph of PL lifetimes, diffusion lifetimes, hole transfer rates, and ionization potential (“HOMO”) offsets. The error bars are defined as errors originated from the instrument and data fitting. **c** Schematic of the behaviors of exciton and charge carriers.

Femtosecond transient absorption (TA) spectroscopy measurements were performed to investigate the interfacial exciton dissociation, which is one of the critical processes in OSCs (Supplementary Figs. 14–17). An 800 nm excitation wavelength was selected to excite only acceptors without exciting donors. In the PM6:BO-4Cl blend, a bleaching peak appears at 855 nm in both the neat BO-4Cl film and the blend film (Supplementary Figs. 16 and 17), which is attributed to the combined contribution of ground state bleaching (GSB) and stimulated emission (SE) of acceptor. The GSB signal of PM6 is located at 570–650 nm based on the TA spectra of the neat PM6 film in Supplementary Fig. 14. In the blend film, following the decay of the acceptor bleach peak at 855 nm, the GSB peak of PM6 appears at ~640 nm and increases in the first 100 ps, which can be assigned to hole transfer from BO-4Cl to PM6. This phenomenon is also observed in the PM6:BO-5Cl and PM6:BO-4Cl:BO-5Cl blends (Supplementary Figs. 15 and  17). Moreover, the hole transfer kinetics extracted from the PM6 GSB signal were shown in Fig. 3a and the bi-exponential fitting of the kinetics yield: *τ*_1_ = 0.236 ± 0.024 and *τ*_2_ = 6.55 ± 0.66 ps in PM6:BO-4Cl; *τ*_1_ = 0.315 ± 0.035 and *τ*_2_ = 23.19 ± 2.32 ps in PM6:BO-5Cl; and *τ*_1_ = 0.265 ± 0.027 and *τ*_2_ = 10.43 ± 1.04 ps in the ternary blend. The fast component *τ*_1_ is commonly assigned to the dissociation of the acceptor exciton formed at the D:A interface, while the second component *τ*_2_ is attributed to diffusion-limited dissociation of bulk excitons^36–38^. Although PM6:BO-4Cl exhibits a faster hole transfer process at the interface, PM6:BO-5Cl presents a larger proportion of the exciton diffusion mediated transfer process and a longer *τ*_2_ over 23 ps, which is associated to the energetic and morphological differences discussed above. The kinetics in the ternary blend are intermediate (*τ*_1_ = 0.265 ± 0.027 and *τ*_2_ = 10.43 ± 1.04 ps) between those of the parent binary blends (*τ*_1_ = 0.236 ± 0.024 and *τ*_2_ = 6.55 ± 0.66 ps in PM6:BO-4Cl, and *τ*_1_ = 0.315 ± 0.035 and *τ*_2_ = 23.19 ± 2.32 ps in PM6:BO-5Cl, respectively), illustrating that the ternary system combines the fast exciton dissociation of PM6:BO-4Cl with the long diffusion process of PM6:BO-5Cl (Fig. 3a, b). Furthermore, by comparing the carrier dynamics of the acceptor GSB in the blend and pristine films (Supplementary Fig. 17) and applying the methodology developed in previous work^37^, we estimate that the exciton dissociation efficiencies in the PM6:BO-4Cl, PM6:BO-5Cl, and ternary blends are 95.8%, 89.4%, and 93.1%, respectively.

To gain a deeper understanding of the hole transfer dynamics from the acceptor excited state to the donor in different systems, we also measured the TA spectra and extracted the kinetics for PM6 blended with BO-4F and BO-6Cl (Supplementary Figs. 16 and 17). The time-resolved photoluminescence (TRPL) results of the neat NFA films vary from 400 to 600 ps (Fig. 3b, Supplementary Fig. 18 and Supplementary Table 6). Thus, the evolution of the exciton diffusion lifetimes (*τ*_2_) measured by the hole transfer process matches well the PL lifetimes of the acceptors, which suggests that longer exciton lifetimes can extend the exciton diffusion lengths and thus the hole transfer times.

Interestingly, the exciton dissociation rate constants (*τ*_1_) as a function of the energetic difference of the D and A ionization potentials ΔIP_D–A_ for all blends shown in Fig. 3b, exhibit a dependence that resembles that of a classical Marcus electron transfer model:

1 $$\frac{1}{{\tau }_{1}}=\frac{2\pi }{{{\hslash }}\sqrt{4\pi \lambda {k}_{\rm {{B}}}T}}{V}^{2}{{{{\rm{{exp }}}}}}\left(-\frac{{\left(\lambda +\varDelta G\right)}^{2}}{4\lambda {k}_{\rm {{B}}}T}\right)$$

where 1/*τ*_1_ represents the hole transfer rate; *k*_B_, the Bolzmann constant; *T*, the temperature; *V*, the electronic coupling between initial and final states; *λ*, the reorganization energy obtained by means of DFT calculations (Supplementary Table 8); and Δ*G*, the driving force for the exciton dissociation given by Δ*E*_LE–CT_ (the difference between the energies of the LE and CT states) or by ΔIP_D–A_ as a simple approximation^39^. Importantly, since the reorganization energies in these blends are calculated to be very small (*λ* ~ 0.09–0.10 eV), the hole transfer rates can remain fast for a ΔIP value as small as, or slightly higher than 0.02 eV; this is a much lower value than the value of ~0.3 eV often quoted as required for the energy offset to drive efficient exciton dissociation^17^. Overall, the Marcus theory model describes well our TA kinetics results.

**Fig. 4:**
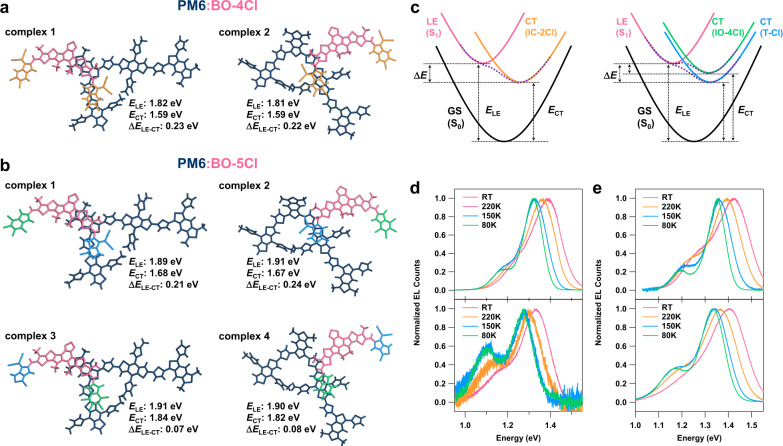
D:A complex configurations, calculated state energies, and temperature-dependent EL spectra. **a** PM6:BO-4Cl and **b** PM6:BO-5Cl complexes as well as the relevant energies of the LE and CT states and their differences. **c** Diagrams of the energy differences. **d** Temperature-dependent EL spectra of BO-4Cl (upper part) and PM6:BO4Cl (lower part) films. **e** Temperature-dependent EL spectra of BO-5Cl (upper part), and PM6:BO-5Cl (lower part) films.

TD-DFT calculations have also been performed on several D:A complexes to characterize the nature of their CT states. The results for PM6:BO-4Cl and PM6:BO-5Cl are collected in Fig. 4a, b, while those for PM6:BO-4F and PM6:BO-6Cl are shown in Supplementary Fig. 23. For the BO-4Cl acceptor, two types of complexes were found depending on whether an IC-2Cl end group is located on top of the PM6 BDD or BDT unit. In the case of BO-5Cl, the results are similar but there appear four types of complexes depending on whether the T-Cl or IO-4Cl end groups of the acceptor interact with the PM6 BDD or BDT moieties. As seen from Fig. 4a, the LE- and CT-state energies are similar for both types of complexes in PM6:BO-4Cl. As a consequence, their Δ*E*_LE–CT_ values are also similar, in the range of 0.22–0.23 eV. In the case of the BO-5Cl blend, the complexes due to the binding of T-Cl groups with PM6 (complexes 1 and 2 in Fig. 4b) are characterized by Δ*E*_LE–CT_ values in the range of 0.21–0.24 eV, which is similar to the values found in PM6:BO-4Cl. However, in the PM6:BO-5Cl complexes where it is the IO-4Cl end group that interacts with PM6 (complexes 3 and 4 in Fig. 4b), the CT-state energies are significantly higher than those in the T-Cl-based complexes; as a result, the Δ*E*_LE–CT_ energies in the IO-4Cl-based complexes are as small as 0.07–0.08 eV. Thus, the asymmetric nature of BO-5Cl allows the formation of a dual interfacial electronic manifold, as shown in Fig. 4c. It remains to investigate whether due to its dual feature the interfacial and transport states in PM6:BO-5Cl are subject to a larger static disorder than the related states in PM6:BO-4Cl. We note that the SCLC data (Supplementary Table 12) indicate the electron mobility in PM6:BO-4Cl is two times larger than in PM6:BO-5Cl, that at first glance might suggest a larger static disorder in the latter blend. However, in the ternary blend the mobility increases back to that in PM6:BO-4Cl, suggesting a much complex situation. Therefore, the work to estimate the energetic disorder and its role on charge recombination and charge transport processes in these binary and ternary blends based on a combination of molecular dynamic simulations and excited-state calculations is currently underway and the results will be reported elsewhere^40,41^.

The Δ*E*_LE–CT_ value is known to determine the extent of LE–CT electronic hybridization and the magnitude of the charge recombination processes^42^. On one hand, the much smaller Δ*E*_LE–CT_ values for the PM6:BO-5Cl complexes 3 and 4 is expected to lead to a stronger hybridization between the CT and LE states. Indeed, as follows from Supplementary Fig. 22, if the electron and hole NTOs in complex 1 are entirely localized on acceptor and donor moieties, respectively, then in complex 3 the hole is delocalized on both donor and acceptor units. The findings that in IO-4Cl-based complexes the CT states are stronger hybridized with the LE states and their energies are about 0.2 eV higher than those in the T-Cl-based complexes (Fig. 4) suggest that they contribute to the observed smaller non-radiative voltage loss in PM6:BO-5Cl blends in comparison to other blends. We anticipate that these states contribute to the observed smaller non-radiative voltage loss in PM6:BO-5Cl blends in comparison to other blends^13^. On the other hand, the complexes formed by the interaction of T-Cl groups with PM6, complexes 1 and 2, provide driving forces for exciton dissociation in PM6:BO-5Cl that are comparable to those provided by the CT states in PM6:BO-4Cl. The combination of those two features highlights that the dual nature of the interfacial structural and electronic characteristics due to the asymmetric design of BO-5Cl can be the fundamentals underlying fast exciton dissociation and small non-radiative voltage losses in both binary and ternary blends.

The TA data, in line with the recent results obtained for PM6:Y6 and other efficient blends^36^, show that in all investigated blends the interfacial excitons dissociate on ultrafast timescales (a few hundred fs). However, due to the contribution coming from exciton diffusion, it takes more than 100 ps to complete the exciton dissociation process^36,43,44^. We note that, in addition to exciton dissociation and exciton diffusion, the TA data might also be affected by other processes such as back electron/hole transfer from CT to LE state^13^, formation of excimers, and decay of CT states to local triplet states^45^. Therefore, in order to shed more light on exciton kinetics and examine the effect of different D:A packing configurations (Fig. 4) on exciton/charge kinetics, we also performed TRPL and electroluminescence (EL) measurements on BO-4Cl and BO-5Cl films and their blends with PM6 (Supplementary Fig. 18). The comparison of the TRPL data shows that the exciton lifetime is larger in BO-5Cl than that in BO-4Cl, when measured in both toluene solution and PS matrix at low acceptor content. We note that the exciton lifetimes measured in PS matrix, 1.49 ns for BO-4Cl and 2.14 ns for BO-5Cl, are comparable to the 1.6 ns lifetime measured for excitons in Y6 films^9^. By making use of the TRPL lifetimes (Supplementary Fig. 18 and Supplementary Table 7) measured for the blends and for BO-4Cl and BO-5Cl in PS matrix, we estimate that the exciton dissociation rate and exciton dissociation efficiency are 19.3 ns^−1^ and 97% in PM6:BO-4Cl, and 7.9 ns^−1^ and 94.0% in PM6:BO-5Cl. These values are in line with those derived from the TA measurements; however, in contrast to the TA estimates, they account for all processes contributing to exciton population decay. We note that rates of a few tens ns^−1^ for exciton population decay were also estimated in other high-performance D:A blends^14^. Finally, we stress that the similar exciton dissociation efficiencies in PM6:BO-4Cl and PM6:BO-5Cl suggest that exciton separation is dominated by complexes 1 and 2 that have larger Δ*E*_LE–CT_ values and faster exciton dissociation rates.

In order to get a better assessment of the Δ*E*_LE-CT_ values, we also measured the temperature-dependent EL spectra of the BO-4Cl and BO-5Cl films and their blends with PM6 (Fig. 4d, e). The EL spectra of both BO-4Cl and BO-5Cl films at 80 K exhibit vibrational satellites with vibrational energies of 130 and 170 meV, respectively. The intensity ratio *I*_2_/*I*_1_ of the second to first peak (i.e., 0–0 to 0–1 vibrational transitions) is about 0.2 in both acceptors. In PM6:BO-4Cl and PM6:BO-5Cl, the *I*_2_/*I*_1_ ratios significantly increase. The energy difference between the first and second peaks in PM6:BO-5Cl resembles that of the acceptor, while in PM6:BO-4Cl it increases to 170 meV. These results strongly suggest that the second EL peak in both blends contains a significant contribution from the CT states. The EL results show that in both blends the *I*_2_/*I*_1_ values decrease with a temperature increase, which confirms the major contribution of the CT states to the second EL peak in the blends. Overall, the low-temperature EL results are consistent with an LE-CT energy difference that does not exceed 160 meV in PM6:BO-5Cl, while it is somewhat larger than 170 meV in PM6:BO-4Cl.

### Photovoltaic properties

**Fig. 5:**
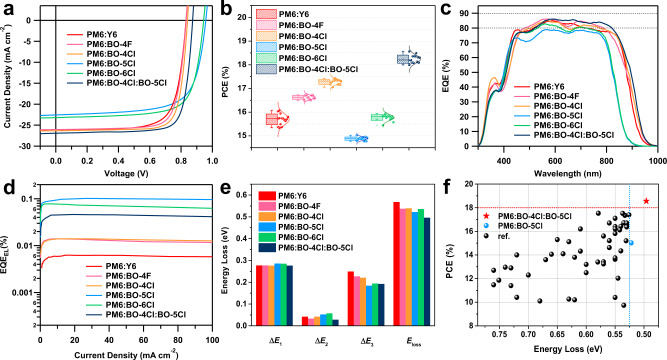
Photovoltaic properties of OSCs. **a** *J*–*V* curves of the optimal devices. **b** PCE statistics (error bar is defined as the standard deviation, which is calculated from the statistics results of 15 devices). **c** EQE curves and **d** EQEEL curves of the optimal devices. **e** Comparison of energy loss in the six types of devices. **f** Comparison of efficiency and energy loss between this work and earlier references (original data in Supplementary Table 16).

The performance of the OSC devices based on PM6:NFA blends was examined by using a conventional device architecture consisting of ITO/PEDOT:PSS/active layer/PFN-Br/Ag. Details of device fabrication and characterizations are provided in the “Methods” section and Supplementary Tables 9–11. The results are summarized in Fig. 5 where the data related to PM6:Y6 are added for the sake of comparison. Figure 5a displays the current density–voltage (*J–V*) curves, with the corresponding photovoltaic parameters summarized in Fig. 5b and Table 1. Compared to PM6:Y6, the *V*_oc_ (0.833 V) and *J*_sc_ (26.04 mA cm^−2^) values of the PM6:BO-4F-based devices are almost unchanged, except that the FF increases from 0.740 to 0.772 due to the improved crystallinity^29^; accordingly, the PCE improves from 16.07% to 16.73%. For the PM6:BO-4Cl based devices, the *V*_oc_ and FF values slightly increase to 0.841 V and 0.794, respectively, yielding a best PCE of 17.43%. For the asymmetric BO-5Cl and BO-6Cl based OSCs, their band gaps are enlarged (Supplementary Table 2) and their *V*_oc_ dramatically increase to 0.958 and 0.944 V, respectively, while efficiencies are in the range of 15–16%. The relatively inferior device performance of the asymmetric BO-5Cl and BO-6Cl with respect to the symmetric BO-4F and BO-4Cl majorly lies in its poor coverage (the formers are about 50 nm blue-shifted than the latters) with solar spectra or the poor *J*_sc_.

**Table 1:** Photovoltaic parameters of devices based on different acceptors.

Active layer	*V*_oc_ (V)	*J*_sc_ (mA cm^−2^)	*J*_cal_ (mA cm^−2^)^a^	FF	PCE (%)^b^
PM6:Y6	0.832 (0.832 ± 0.003)	26.04 (25.93 ± 0.24)	25.70	0.740 (0.728 ± 0.010)	16.07 (15.69 ± 0.22)
PM6:BO-4F	0.833 (0.832 ± 0.002)	26.04 (26.04 ± 0.15)	25.85	0.772 (0.768 ± 0.003)	16.73 (16.62 ± 0.08)
PM6:BO-4Cl	0.841 (0.841 ± 0.002)	26.03 (25.87 ± 0.20)	25.82	0.794 (0.792 ± 0.003)	17.43 (17.28 ± 0.10)
PM6:BO-5Cl	0.958 (0.957 ± 0.001)	22.57 (22.47 ± 0.08)	21.92	0.701 (0.695 ± 0.002)	15.02 (14.88 ± 0.07)
PM6:BO-6Cl	0.944 (0.944 ± 0.001)	23.22 (23.09 ± 0.19)	22.68	0.729 (0.725 ± 0.004)	15.94 (15.77 ± 0.12)
PM6:BO-4Cl:BO-5Cl	0.874 (0.872 ± 0.002)	26.93 (26.65 ± 0.27)	26.63	0.788 (0.784 ± 0.005)	18.56 (18.24 ± 0.16)
PM6:BO-4Cl:BO-5Cl	0.865	26.88	–	0.782	18.2^c^

^a^Integrated current densities from EQE curves.

^b^Average PCEs from 15 devices.

^c^Certified by National Institute of Metrology (NIM), China.

As shown in the EQE spectra of the four NFA-based devices in Fig. 5c, all NFA-based active layers exhibit high EQE values (~80%), which points to efficient internal carrier conversion (or charge transfer/collection). The EQEs in the asymmetric-acceptor devices exhibit a blue-shift by about 70 nm (0.11 eV) compared to the symmetric cases, which is consistent with the observed decrease in *J*_sc_ in the former devices. The photovoltaic bandgaps (*E*_g_^PV^) are estimated to be 1.46, 1.47, 1.45, 1.56, 1.55, and 1.44 eV for PM6:Y6, PM6:BO-4F, PM6:BO-4Cl, PM6:BO-5Cl, PM6:BO-6Cl, and PM6:BO-4Cl:BO-5Cl, respectively, based on the method developed by Rau et al. (Supplementary Fig. S24)^46,47^. When integrating the EQE spectra with the solar spectrum (AM 1.5 G 1 sun), we find good correspondence between the integrated *J*_cal_ values and the *J*_sc_ from the *J–V* curves (Table 1).

The major sources of *V*_oc_ losses in OSCs can be characterized through the energy losses (*E*_loss_) coming from (i) charge recombination from the unavoidable black body radiation (leading to the Shockley–Queisser limit, ∆*E*_1_), (ii) non-ideal radiative decay (∆*E*_2_, radiative loss from below-gap absorption), and (iii) non-radiative decay (∆*E*_3_)^48–50^. A detailed *E*_loss_ analysis was carried out as shown in Fig. 5d, e and Table 2 (the methodology applied for the *E*_loss_ analysis can be found in the Supplementary Fig. 24). There is no significant difference in terms of ∆*E*_1_ due to the similar energy gaps of the five NFA-based devices. For the non-ideal radiative loss, PM6:Y6, PM6:BO-4F, PM6:BO-4Cl, PM6:BO-5Cl, and PM6:BO-6Cl exhibit different values of 0.042, 0.033, 0.042, 0.052, and 0.057 eV, respectively. The somewhat larger non-ideal radiative loss of the asymmetric acceptors can be attributed to the greater variation in molecular packing configurations between the asymmetric molecules in the amorphous state and/or between the donors and the acceptors, as shown by the DFT calculations.

**Table 2:** Detailed energy losses in devices based on different acceptors.

Active layer	*E*_g_^PV^ (eV)	*qV*_oc_ (eV)	*E*_loss_ (eV)	$q{V}_{{{\rm {oc}}}}^{{{\rm {SQ}}}}$ (eV)	$q{V}_{{{\rm {oc}}}}^{{{\rm {rad}}}}$ (eV)	∆*E*_1_ (eV)	∆*E*_2_ (eV)	∆*E*_3_ (eV)	EQE_EL_ (10^−4^)	Exp. $q\triangle {V}_{{{\rm {oc}}}}^{{{\rm {non}}}-{{\rm {rad}}}}$ (eV)
PM6:Y6	1.40	0.832	0.568	1.123	1.081	0.277	0.042	0.249	0.6	0.250
PM6:BO-4F	1.37	0.833	0.537	1.093	1.060	0.277	0.033	0.227	1.3	0.231
PM6:BO-4Cl	1.38	0.841	0.539	1.104	1.062	0.276	0.042	0.221	1.4	0.229
PM6:BO-5Cl	1.48	0.958	0.522	1.194	1.142	0.286	0.052	0.184	10.2	0.178
PM6:BO-6Cl	1.48	0.944	0.536	1.195	1.138	0.285	0.057	0.194	7.2	0.187
PM6:BO-4Cl:BO-5Cl	1.37	0.874	0.496	1.094	1.066	0.276	0.028	0.192	4.6	0.198

The most striking difference occurs when comparing the EQE_EL_ results (Fig. 5d and Table 2). Importantly, the EQE_EL_ values of 6.2 × 10^−5^, 1.3 × 10^−4^, and 1.4 × 10^−4^, corresponding to symmetric Y6, BO-4F, and BO-4Cl based blends are substantially smaller than the values of 1.02 × 10^−3^ and 7.2 × 10^−4^ measured for asymmetric BO-5Cl and BO-6Cl, respectively. These EQE_EL_ values result in non-radiative voltage losses of 0.250, 0.231, 0.229, 0.178, and 0.187 V for the Y6, BO-4F, BO-4Cl, BO-5Cl, and BO-6Cl based devices. Notably, the low non-radiative energy loss of 0.178 eV in the BO-5Cl case is one of the lowest among high-performance OSCs, we attribute it to the high luminescence efficiency of the PM6:BO-5Cl blend and the small energy offset between the LE and CT states as described in the previous section. The combination of the high EQE_EL_ of 0.1% and large EQE_PV_ of 80% in the BO-5Cl-based device strikes the right balance between non-radiative charge recombination and charge generation. The device with the asymmetric BO-5Cl acceptor exhibits the lowest total *V*_oc_ loss of 0.522 eV, which represents one of the lowest values among high-performance organic solar cells. as shown in Fig. 5f.

Remarkably, the highly luminescent behavior of BO-5Cl can be exploited in other systems, specifically by making BO-5Cl a component in a ternary blend. For example, when blending 20% wt BO-5Cl into PM6:BO-4Cl, the charge recombination centers remain at the PM6:BO-4Cl interface, since the EL spectra of the ternary device keep a shape similar to that in PM6:BO-4Cl (Supplementary Fig. 25). However, the ternary device has a significantly improved EQE_EL_, by a factor of 3 compared to the PM6:BO-4Cl binary system (Fig. 5d). This results in a much-reduced non-radiative loss Δ*E*_3_ from 0.229 to 0.198 eV. In addition, the non-ideal radiative loss Δ*E*_2_ goes down to 0.028 eV upon adding BO-5Cl to the active layer. The synergistic effect coming from the decrease in non-ideal radiative and non-radiative contributions leads to a significantly reduced *E*_loss_, from 0.539 to 0.496 eV in the ternary blend, which yields a major increase in *V*_oc_ by 33 mV (Fig. 5a and Table 1). We stress that the *E*_loss_ value in the ternary blend represents one of the lowest among all high-performance organic solar cells (Fig. 5f). Besides, as shown in Fig. 5c, higher EQE response in the acceptor absorbing region (600–850 nm) was obtained in ternary blends compared with PM6:BO-4Cl binary blends, leading to the *J*_sc_ increase, which can be attributed to the improved charge generation in this region with the introduction of BO-5Cl. Considering the high EQE and FF values, the ternary device incorporating BO-5Cl delivers an outstanding balance among non-radiative decay suppression, efficient charge generation, and transport. The best ternary device exhibits a high PCE of 18.56%, with certified value 18.2% by National Institute of Metrology (NIM), China. This result is among the very highest reported certified PCEs to date. The introduction of asymmetric BO-5Cl results in more diverse D:A intermolecular conformations, which will balance the charge generation and charge recombination. Besides of the dilution effect^6^, the ternary blends (polymer donor with two different acceptors) could also construct diverse molecular conformations when an asymmetric molecule is added as the third component, which might show the similar effect as the asymmetric molecule appearing in the binary blend. Our findings validate the rationale of using asymmetric acceptors to achieve high luminescence and faster charge transfer for high-performance ternary-blend organic photovoltaic devices.

We have demonstrated that the use of asymmetric acceptors offers a remarkable balance between charge transfer and charge recombination. We fabricated highly luminescent, efficient organic photovoltaic devices based on the PM6 donor and the BO-5Cl asymmetric acceptor, which is due to a better balance between the charge generation (EQE and *J*_sc_) and charge recombination (*V*_oc_ or *V*_oc_ loss) formed by more diverse D:A intermolecular conformations. Also, we discovered that the high-luminescence behavior can be transferred into other systems by developing ternary blends. In this instance, a high PCE of 18.56% is obtained (certified as 18.2%), which is due to a major reduction in *V*_oc_ loss. These devices are among those with the very highest reported certified PCE values. Our joint experimental and theoretical efforts underline that the origin of the efficiency of organic solar cells based on asymmetric acceptors lies in the duality of the specific packing configurations and electronic characteristics at the D:A interfaces, which contribute to a better balance between charge generation and recombination in both binary and ternary active layers.

## Methods

### Device fabrication

Organic solar cells were fabricated on glass substrates commercially pre-coated with a layer of indium tin oxide (ITO), based on the inverted structure: ITO/PEDOT:PSS/active layer/PFN-Br/Ag. Before fabrication, the substrates were consecutively pre-cleaned by an ultrasonic bath of detergent, deionized water, acetone, and isopropanol, and then treated in an ultraviolet ozone generator for 15 min. Then, a thin PEDOT:PSS (Baytron P AI4083) layer was spin coated onto the substrates at 4500 rpm (~20 nm thick) for 30 s and annealed at 170 °C for 20 min. The substrates were placed into a glovebox and all active layers were spin coated from 17.6 mg mL^−1^ (PM6:acceptor = 1:1.2) chloroform solution at 3000 rpm for 30 s with different additives. Detailed device fabrication conditions are summarized in Supplementary Tables 9–11. Then, a 5 nm PFN-Br film was deposited as the cathode buffer layer by spin-coating of a solution of 0.5 mg mL^−1^ PFN-Br in methanol. Finally, the Ag (100 nm) electrode was deposited by thermal evaporation. The device was completed with an active area of 0.0925 cm^2^, as defined by the overlapping area of ITO and Ag.

### *J–V* and EQE measurements

The current density–voltage (*J–V*) curves of OSCs were measured on an Enlitech SS-F5-3A solar simulator under AM 1.5 G illumination, whose light intensity was calibrated by a standard Si solar cell at 100 mV cm^−2^. Devices were tested in N_2_-filled glove box. The scan direction is −0.2 to 1.2 V, with a scan step of 0.01 V and dwell time is 1 ms. The EQE data were measured by a Solar Cell Spectral Response Measurement System (RE-R, Enlitech). All of the devices mentioned were tested through a shadow mask with an area of 0.05979 cm^2^ (certified by National Institute of Metrology, China).

### Reporting summary

Further information on research design is available in the Nature Research Reporting Summary linked to this article.

## Supplementary information

Supplementary Information

Solar Cells Reporting Summary

## Data Availability

The data that support the plots within this paper and other finding of this study are available from the corresponding authors upon reasonable request. CCDC number 2108163, 2108164, 2113878 contain the supplementary crystallographic data for this paper. These data can be obtained free of charge from the Cambridge Crystallographic Data Centre via www.ccdc.cam.ac.uk/data_request/cif.
