# Supplementary material for: Asymmetric electron acceptor enables highly luminescent organic solar cells with certified efficiency over 18%
Source: Nat Commun. 2022 May 11;13:2598. doi: 10.1038/s41467-022-30225-7 (PMC9095617; doi:10.1038/s41467-022-30225-7)
Supplement: Supplementary file 1 — Supplementary Information [file 41467_2022_30225_MOESM1_ESM.pdf]

Supplementary Information

Asymmetric electron acceptor enables highly luminescent organic solar cells with certified efficiency over 18%

Chengliang He[1,9], Zeng Chen[2,9], Tonghui Wang[3,9], Ziqiu Shen[1], Yaokai Li[1], Jiadong Zhou[4], Jianwei Yu[5], Huiyu Fang[6], Yuhao Li[7], Shuixing Li[1], Xinhui Lu[7], Wei Ma[6], Feng Gao[5], Zengqi Xie[4], Veaceslav Coropceanu[3]*, Haiming Zhu[2]*, Jean-Luc Bredas[3], Lijian Zuo[1,8]* & Hongzheng Chen[1]*

[1]State Key Laboratory of Silicon Materials, MOE Key Laboratory of Macromolecular Synthesis and Functionalization, Department of Polymer Science and Engineering, Zhejiang University, Hangzhou 310027, P.R. China.

[2]Department of Chemistry, Zhejiang University, Hangzhou 310027, P.R. China.

[3]Department of Chemistry and Biochemistry, The University of Arizona, Tucson, Arizona 85721-0088, USA.

[4]Institute of Polymer Optoelectronic Materials and Devices, State Key Laboratory of Luminescent Materials and Devices, South China University of Technology, Guangzhou 510640, P.R. China.

[5]Department of Physics, Chemistry, and Biology, Linköping University, Linköping SE-58183, Sweden.

[6]State Key Laboratory for Mechanical Behavior of Materials, Xi'an Jiaotong University, Xi'an 710049, P.R. China.

[7]Department of Physics, Chinese University of Hong Kong, New Territories, Hong Kong 999077, P. R. China.

[8]Zhejiang University-Hangzhou Global Scientific and Technological Innovation Center, Hangzhou 310014, P. R. China.

[9]These authors contributed equally: Chengliang He, Zeng Chen, Tonghui Wang.

*E-mail: coropceanu@arizona.edu; hmzhu@zju.edu.cn; zjuzlj@zju.edu.cn; hzchen@zju.edu.cn.

Supplementary Methods

Materials Synthesis

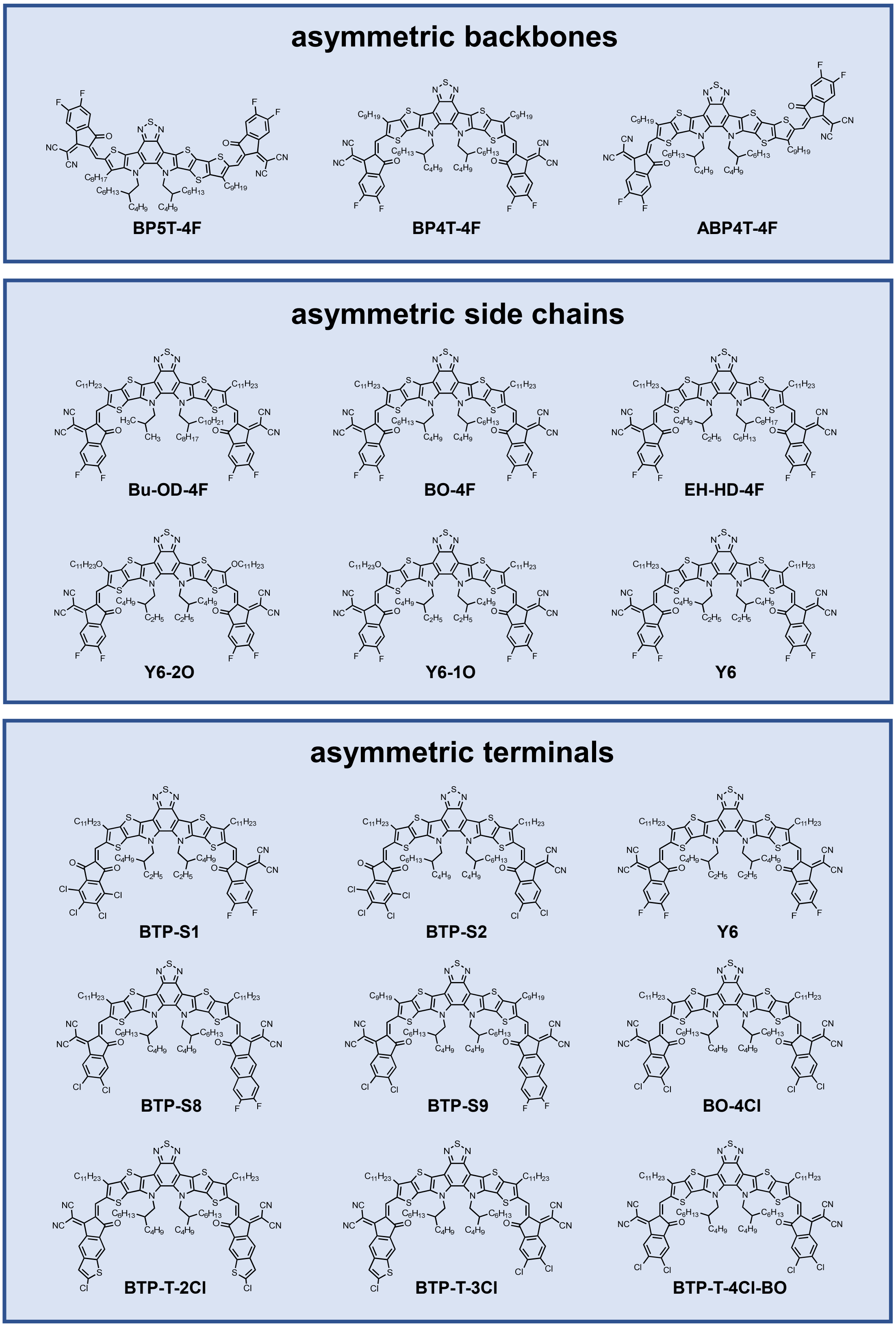

Supplementary Fig. 1 | Chemical structures of summarized Y-series asymmetric acceptors.

All reagents and solvents, unless otherwise specified, were purchased from commercial sources and were used without further purification. PM6 is purchased from Solarmer Material Inc. BTP-2CHO is purchased from Derthon Optoelectronics Materials Science Technology Co LTD. BO-4F, BO-4Cl and BO-6Cl were synthesized in our lab according to the methods in literatures. [1,2] The synthetic routes of these acceptors are provided in **Supplementary Fig. 2**.

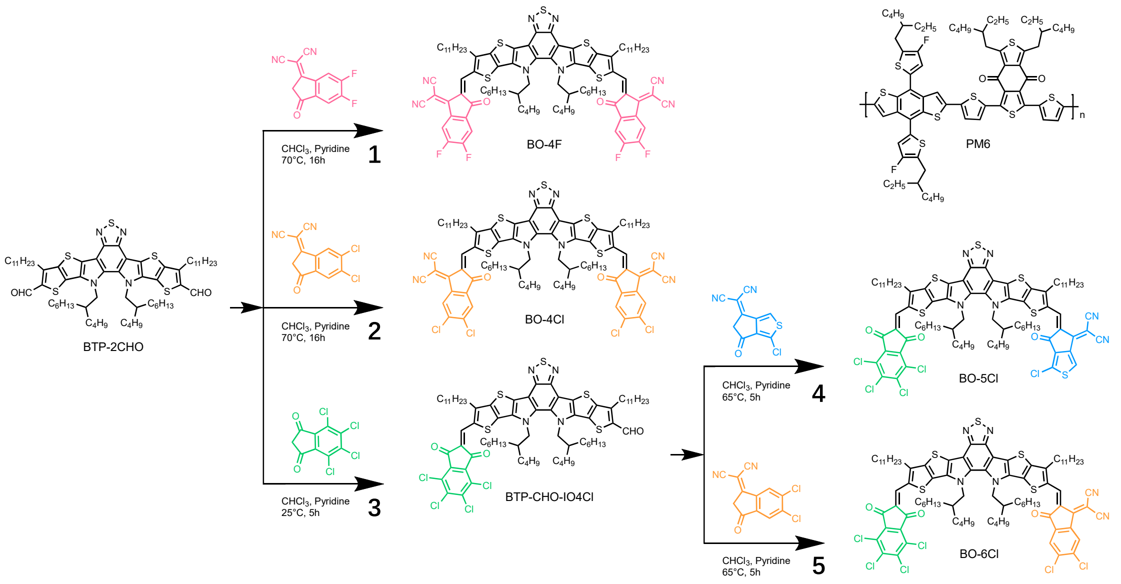

Supplementary Fig. 2 | Synthetic routes for the acceptors and chemical structure of the polymer donor.

(Z)-2-(5-((12,13-bis(2-butyloctyl)-10-((4,5,6,7-tetrachloro-1,3-dioxo-1,3-dihydro-2H-inden-2-ylidene)methyl)-3,9-diundecyl-12,13-dihydro-[1,2,5]thiadiazolo[3,4-e]thieno[2'',3'':4',5']thieno[2',3':4,5]pyrrolo[3,2-g]thieno[2',3':4,5]thieno[3,2-b]indol-2-yl)methylene)-1-chloro-6-oxo-5,6-dihydro-4H-cyclopenta[c]thiophen-4-ylidene)malononitrile (BO-5Cl)

To a Schlenk tube were added compound BTP-CHO-IO4Cl (0.15 g, 0.106 mmol), 2-(1-chloro-6-oxo-5,6-dihydro-4H-cyclopenta[c]thiophen-4-ylidene)malononitrile (0.062 g, 0.265 mmol) and dried $CHCl_3$ (30 mL). The mixture was frozen in liquid nitrogen, followed by three times of successive vacuum and Ar gas filling cycles. Then 0.5 mL pyridine was added. The mixture was refluxed at 65 °C for 5 h. After removing the solvent, silica gel column chromatography was used to purify the product with the mixture of petroleum ether and dichloromethane (3:1~2:1, v/v) as the eluent, yielding a black solid (0.14 g, 81%). ^{1}H NMR (400 MHz, $CDCl_3$, δ): 9.07 (s, 1H), 8.26 (s, 1H), 8.16 (s, 1H), 4.90-4.65 (m, 4H), 3.24 (q, J = 7.0 Hz, 4H), 2.25-2.07 (m, 2H), 1.97-1.78 (m, 4H), 1.54-1.44 (m, 4H), 1.42-1.33 (m, 4H), 1.33-1.22 (m, 28H), 1.18-0.83 (m, 34H), 0.73-0.62 (m, 12H). MS (MALDI-TOF): calculated for $C_{85}H_{99}Cl_5N_6O_3S_6$ (M^+): 1622.38, found:1622.01.

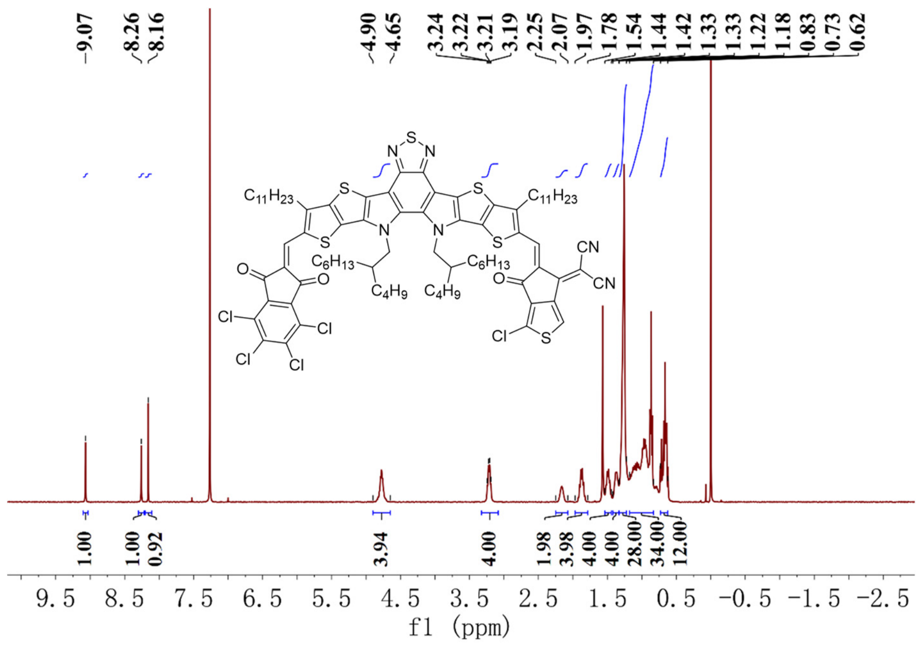

Supplementary Fig. 3 | NMR spectrum of BO-5Cl.

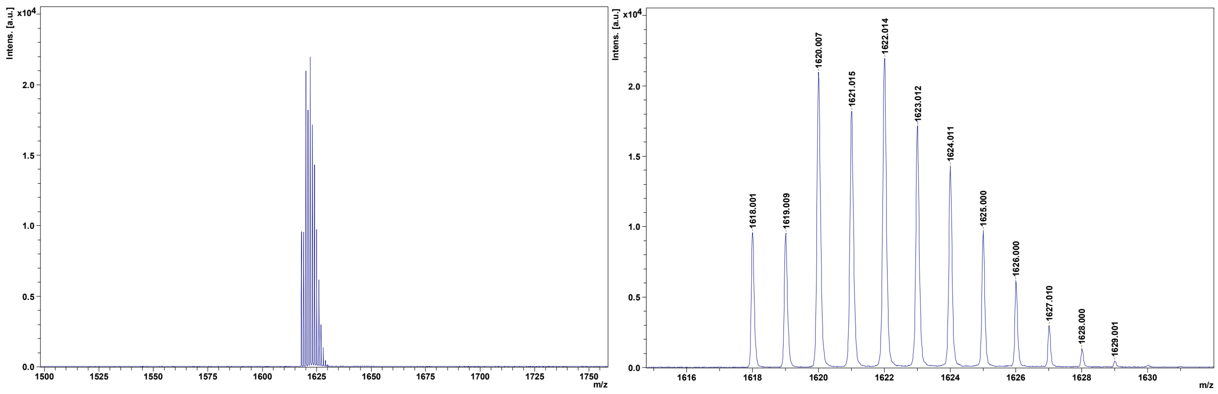

Supplementary Fig. 4 | High-resolution mass spectrum of BO-5Cl.

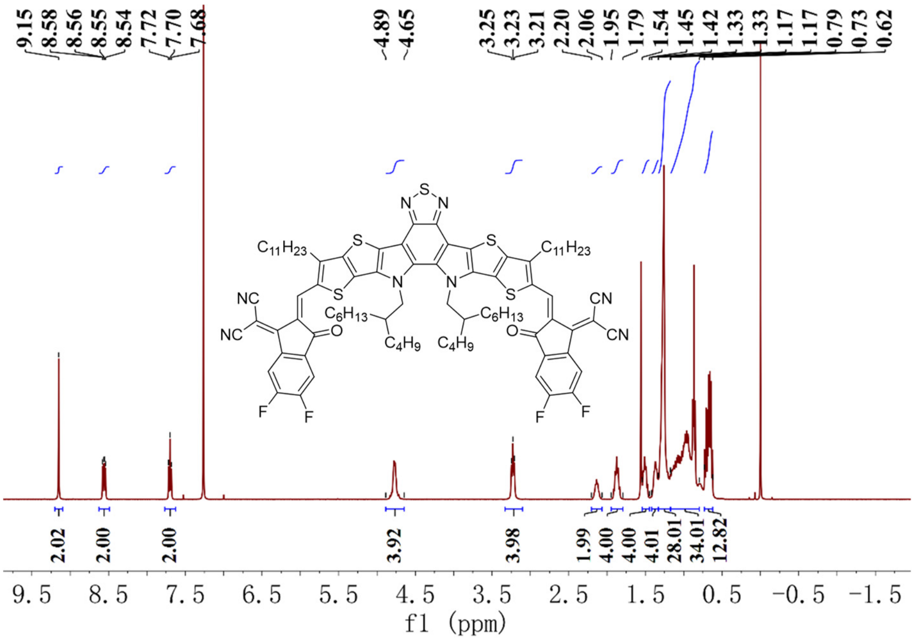

Supplementary Fig. 5 | NMR spectrum of BO-4F.

BO-4F ^{1}H NMR (400 MHz, $CDCl_3$, δ): 9.15 (s, 2H), 8.58 (q, J = 6.5 Hz, 2H), 8.58 (t, J = 7.5 Hz, 2H), 4.89-4.65 (m, 4H), 3.25 (t, J = 7.6 Hz, 4H), 2.20-2.06 (m, 2H), 1.95-1.79 (m, 4H), 1.54-1.45 (m, 4H), 1.42-1.33 (m, 4H), 1.33-1.17 (m, 28H), 1.17-0.79 (m, 34H), 0.73-0.62 (m, 12H).

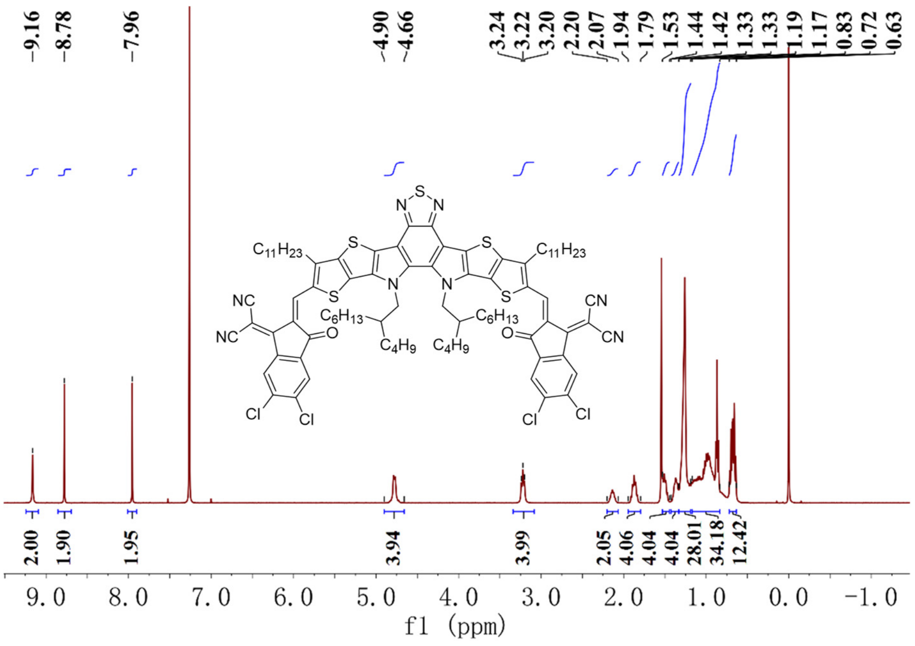

Supplementary Fig. 6 | NMR spectrum of BO-4Cl.

BO-4Cl ^{1}H NMR (400 MHz, $CDCl_3$, δ): 9.16 (s, 2H), 8.78 (s, 2H), 7.96 (s, 2H), 4.90-4.66 (m, 4H), 3.24 (t, J = 7.6 Hz, 4H), 2.20-2.07 (m, 2H), 1.94-1.79 (m, 4H), 1.53-1.44 (m, 4H), 1.42-1.33 (m, 4H), 1.33-1.19 (m, 28H), 1.17-0.83 (m, 34H), 0.72-0.63 (m, 12H).

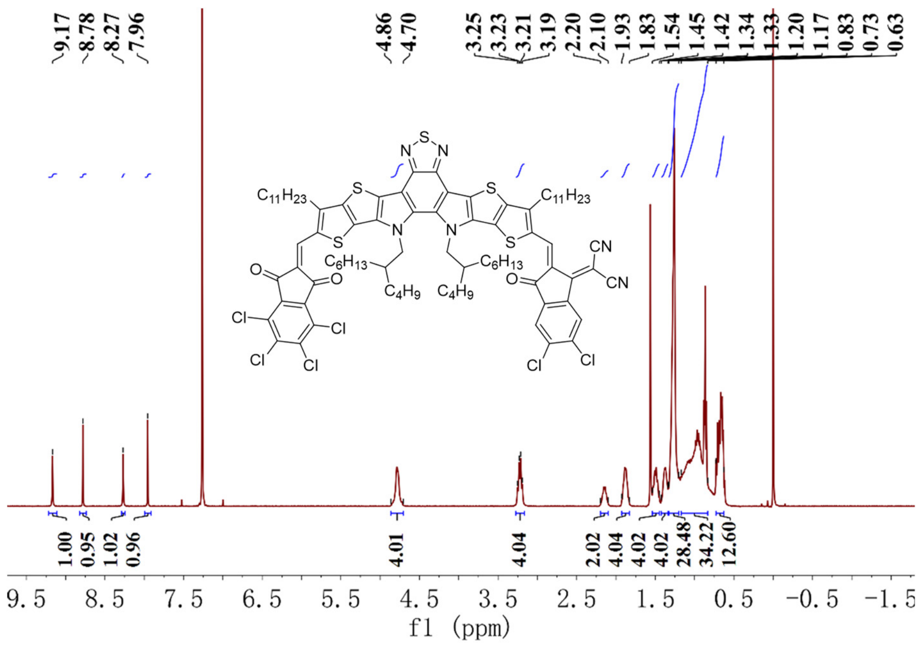

Supplementary Fig. 7 | NMR spectrum of BO-6Cl.

BO-6Cl ^{1}H NMR (400 MHz, $CDCl_3$, δ): 9.17 (s, 1H), 8.78 (s, 1H), 8.27 (s, 1H), 7.96 (s, 1H), 4.86-4.70 (m, 4H), 3.25 (q, J = 8.7 Hz, 4H), 2.20-2.10 (m, 2H), 1.93-1.83 (m, 4H), 1.54-1.45 (m, 4H), 1.42-1.34 (m, 4H), 1.33-1.20 (m, 28H), 1.17-0.83 (m, 34H), 0.73-0.63 (m, 12H).

^{1}H NMR spectra were carried out with a Bruker Advance III 400 (400 MHz) nuclear magnetic resonance (NMR) spectroscope. Matrix-assisted laser desorption/ionization time of flight (MALDI-TOF) MS spectra were obtained on the Bruker Ultra-flex MALDI.

UV-vis Absorption Spectroscopy and Photoluminescence (PL) Spectra

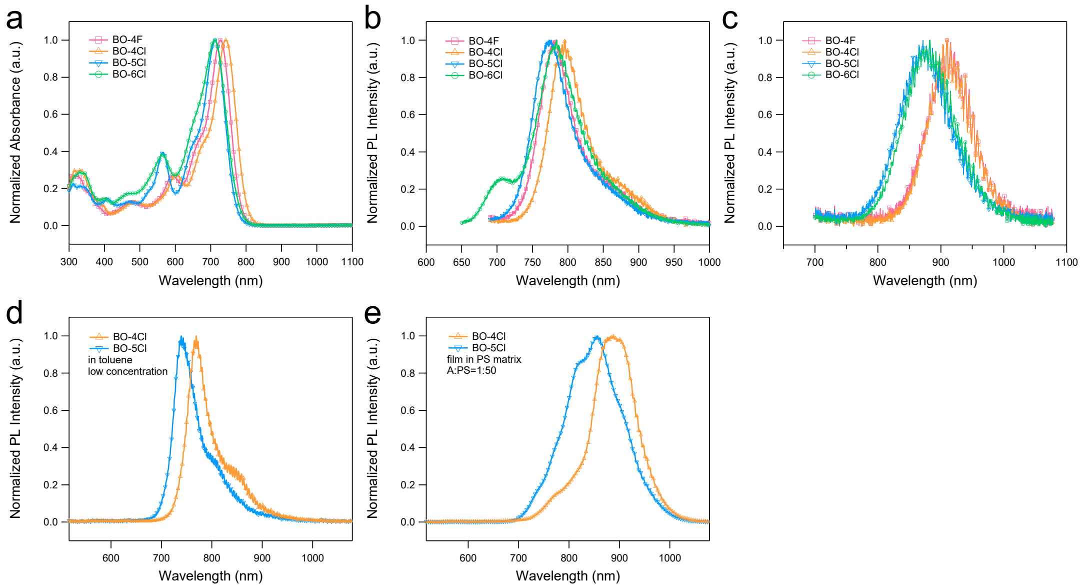

Supplementary Fig. 8 | a Normalized absorption spectra of the acceptors in chloroform solution. Normalized PL spectra of the acceptors in **b** chloroform solution and **c** thin films. **d** PL spectra of the acceptors in toluene at low concentration (0.05 mg/mL). **e** PL spectra of the acceptors blended in polystyrene (PS) at the 1:50 mass ratio.

UV-vis absorption spectra were measured on a Shimadzu UV-1800 spectrophotometer.

Ultraviolet Photoelectron Spectroscopy (UPS) and Cyclic Voltammetry (CV)

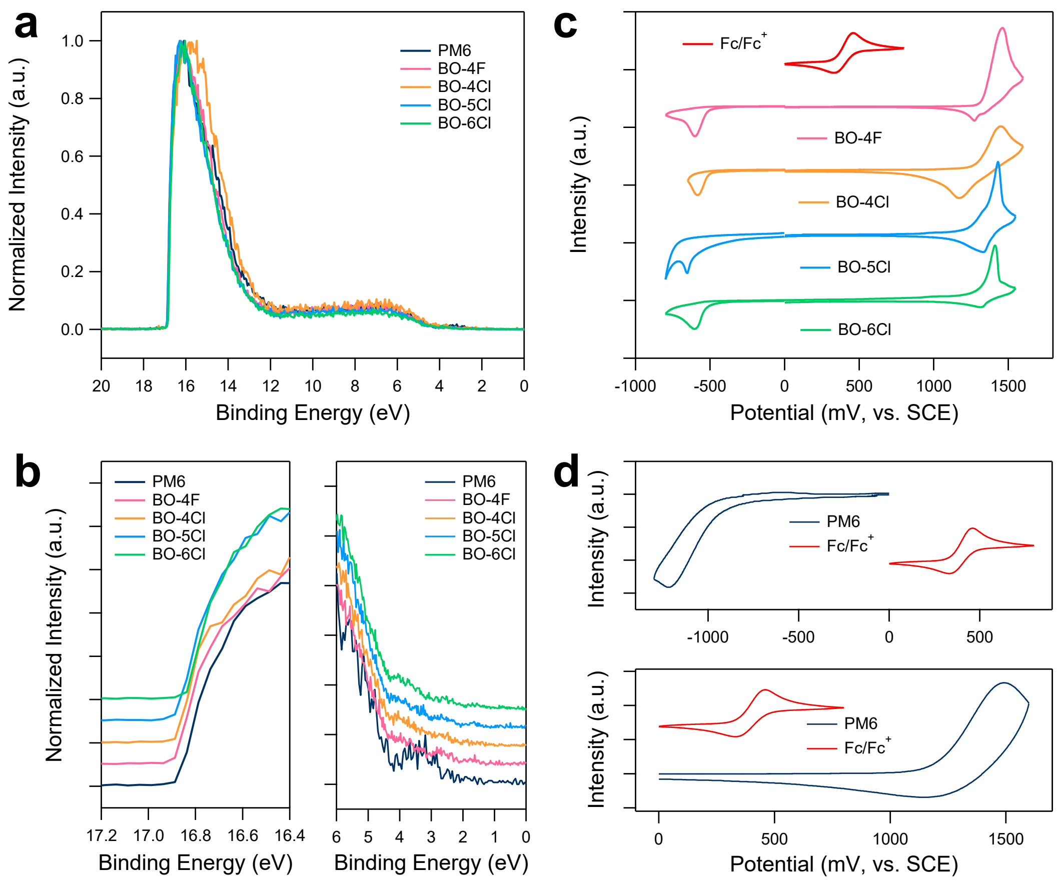

Supplementary Fig. 9 | a,**b** UPS (He I; hν = 21.22 eV) data of neat films. **c**,**d** Cyclic voltammograms.

Cyclic voltammetry was done on a CHI600A electrochemical workstation by utilizing the acetonitrile solution of 0.1 mol/L tetrabutylammoniumhexafluorophosphate (Bu_4NPF_6). The CV curves were recorded versus the potential of SCE, which was calibrated by the ferrocene-ferrocenium (Fc/Fc^+) redox couple (4.8 eV below the vacuum level). Then LUMO and HOMO levels was calculated by the equation:

$$E_{LUMO/HOMO} = -e\left(E_{red/ox} + 4.41\right)\left(eV\right) \quad (1)$$

Single-Crystal Analysis

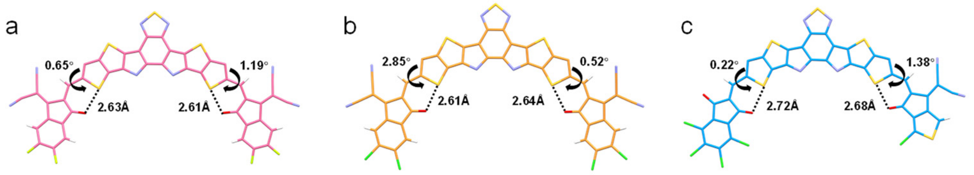

Supplementary Fig. 10 | Single-crystal molecular structures of **a** BO-4F, **b** BO-4Cl and **c** BO-5Cl.

Crystal Growth - Solvent diffusion. A solution prepared from ~ 5 mg NFA in 0.5 mL $CHCl_3$ was transferred into a cylindrical tube with a diameter of 8 mm. Around 2 mL of methanol was carefully layered on top of the $CHCl_3$ solution. The tube was then sealed and left standing for a few days (7-10 days) until the color of the solution faded away. Single crystal data were collected on Rigaku XtaLAB P2000 FR-X at 100 K.

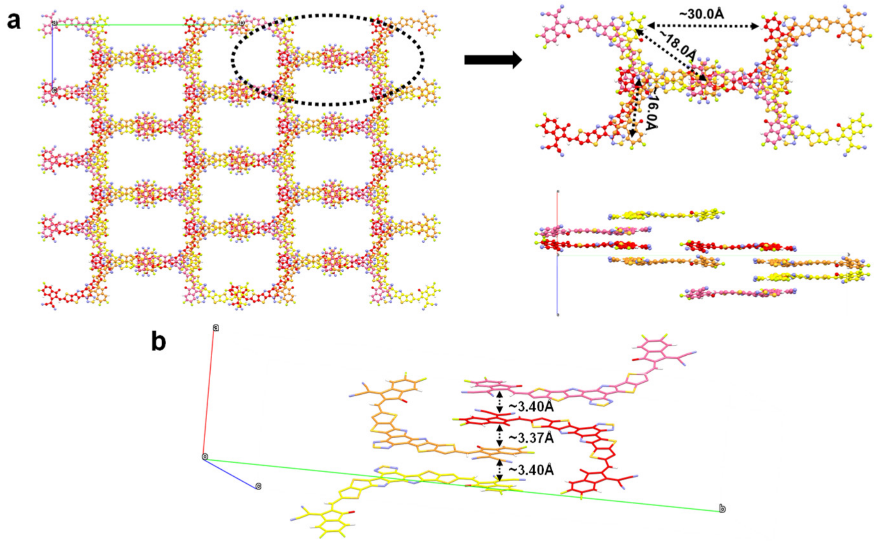

Supplementary Fig. 11 | Molecular stacking patterns of BO-4F in the single-crystal structure.

GIWAXS Images

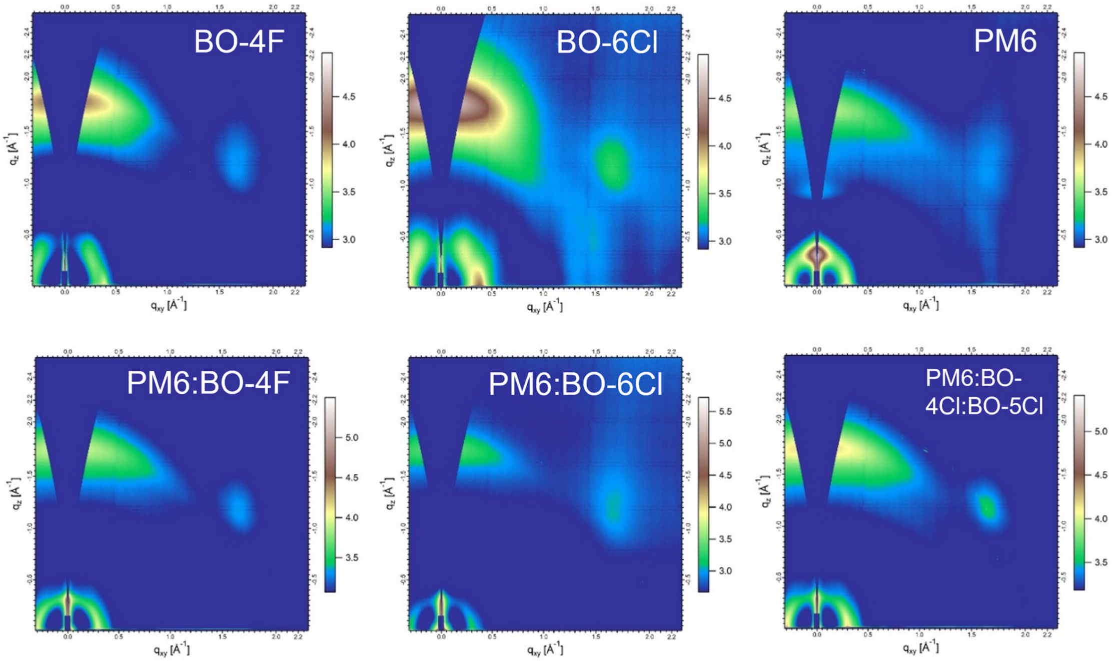

Supplementary Fig. 12 | GIWAXS images of the pristine films and blend films.

GIWAXS measurements were performed at beamline 7.3.3 at the Advanced Light Source. Samples were prepared on Si substrates using identical blend solutions as those used in devices. The 10 keV X-ray beam was incident at a grazing angle of 0.11°-0.15°, selected to maximize the scattering intensity from the samples. The scattered x-rays were detected using a Dectris Pilatus 2M photon counting detector.

GISAXS Images

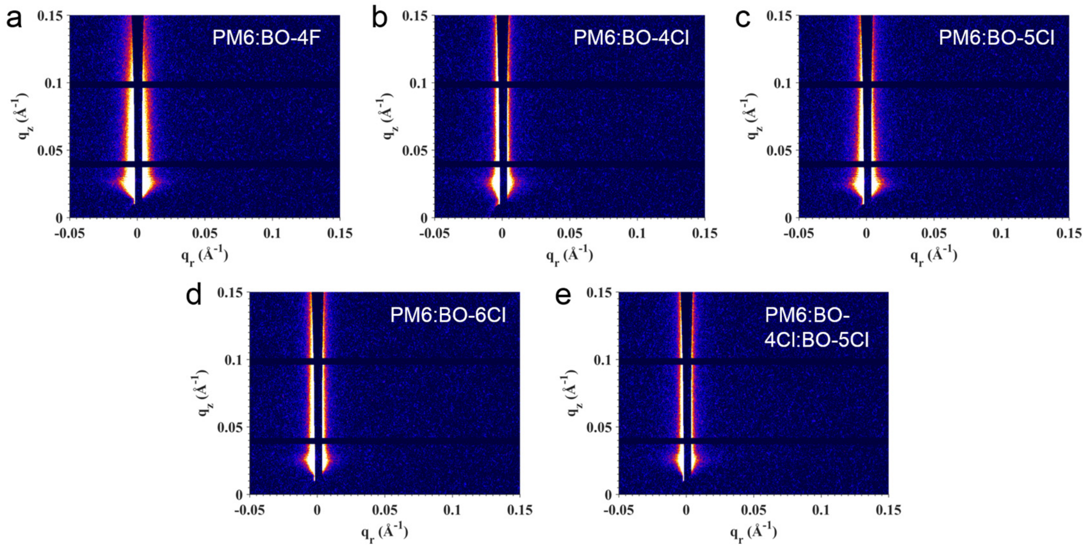

Supplementary Fig. 13 | GISAXS images of the blends.

GISAXS measurements were carried out at BL19U2 in the Shanghai Synchrotron Radiation Facility. The wavelength is 1.03 Å. The incidence angle is 0.1°.

Femtosecond Transient Absorption Spectroscopy (TAS)

To carry out femtosecond transient absorption spectroscopy, the fundamental output from a Yb:KGW laser (1030 nm, 220 fs Gaussian fit, 100 kHz, Light Conversion Ltd) was separated into two light beams. One was introduced into a NOPA (ORPHEUS-N, Light Conversion Ltd) to produce a specific wavelength for the pump beam (here, we used 750 nm), the other was focused onto a YAG plate to generate a white light continuum as the probe beam. The pump and probe overlapped on the sample at a small angle lesser than 10°. The probe light transmitted by the sample was collected by a linear CCD array. Then, we obtained the transient differential transmission signals according to the following equation:

$$\frac{\Delta T}{T} = \frac{T_{pump-on} - T_{pump-off}}{T_{pump-off}} \tag{2}$$

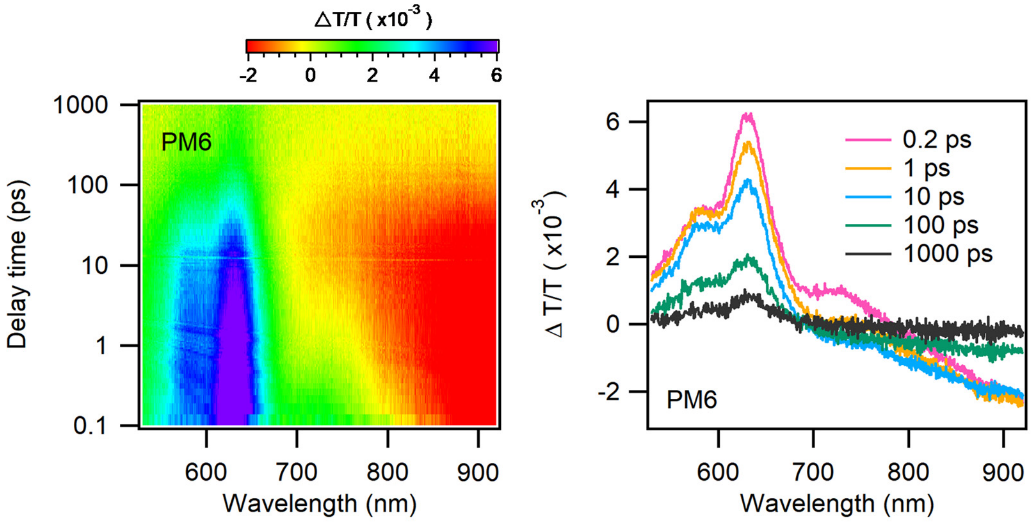

Supplementary Fig. 14 | Color plot and dynamic curves of the TA result for PM6.

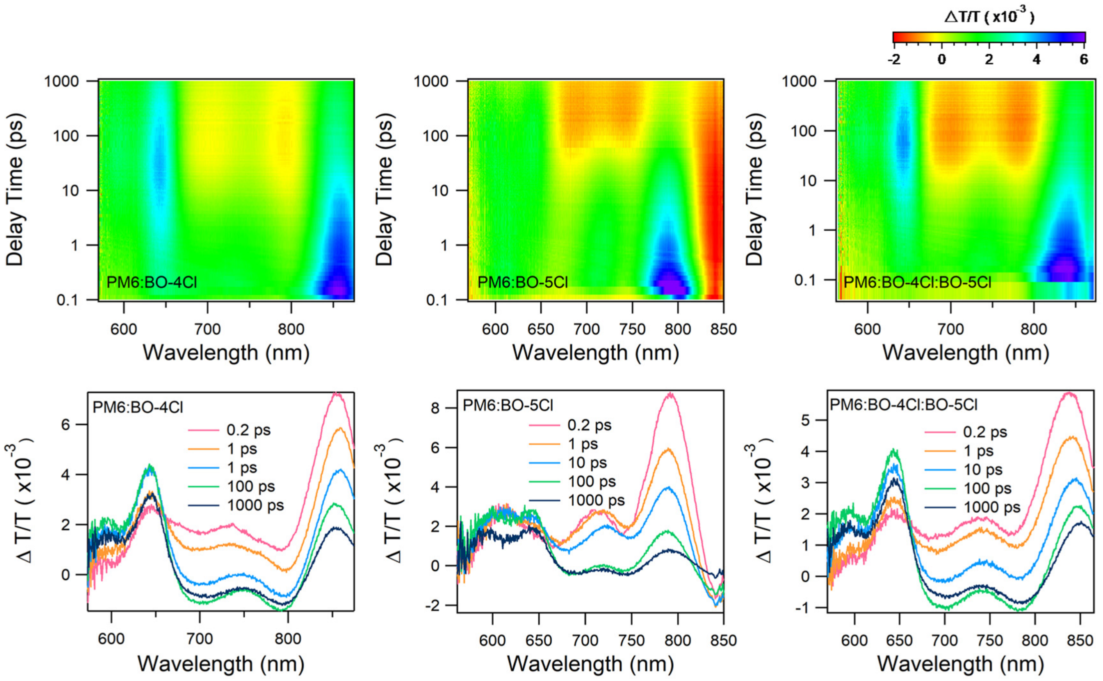

Supplementary Fig. 15 | Color plots and dynamic curves of the TA results of the corresponding blend films.

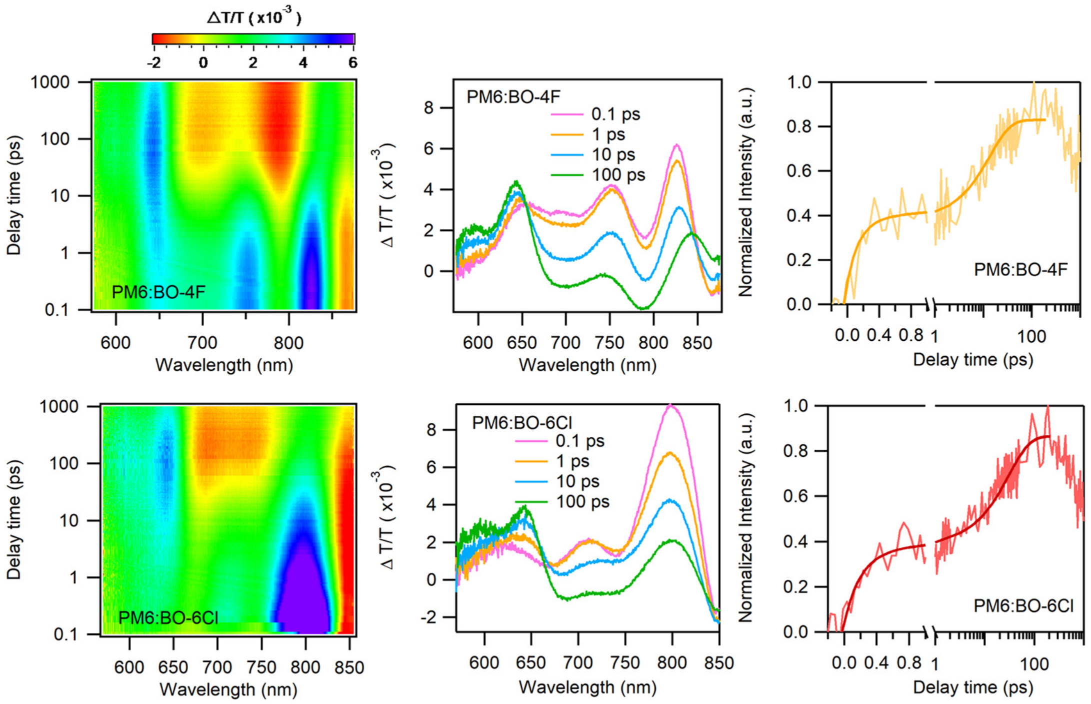

Supplementary Fig. 16 | Color plots and dynamic curves of the TA results of the corresponding blend films.

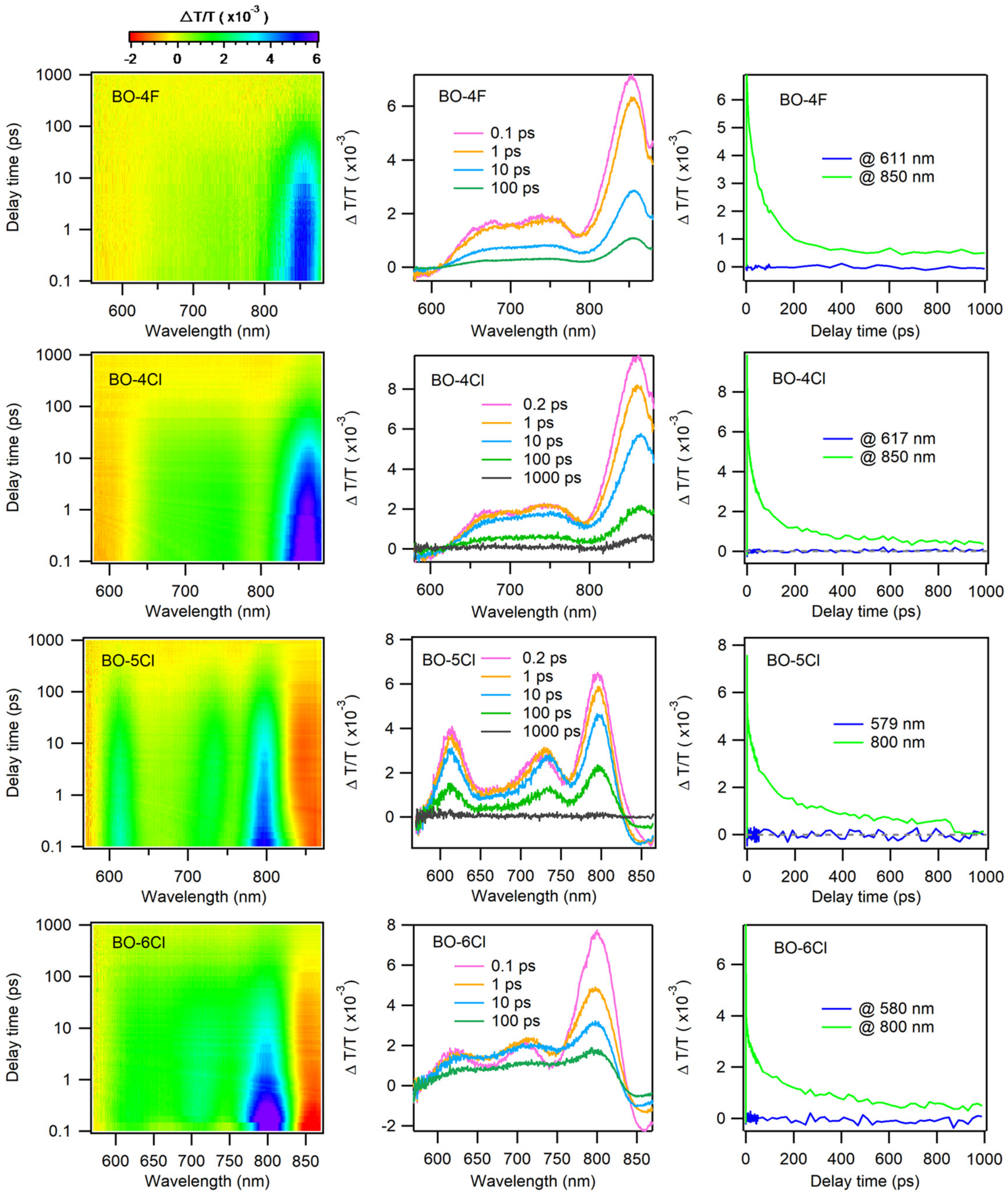

Supplementary Fig. 17 | Color plots and dynamic curves of the TA results for the pure acceptors.

Time-Resolved Photoluminescence (TRPL) results

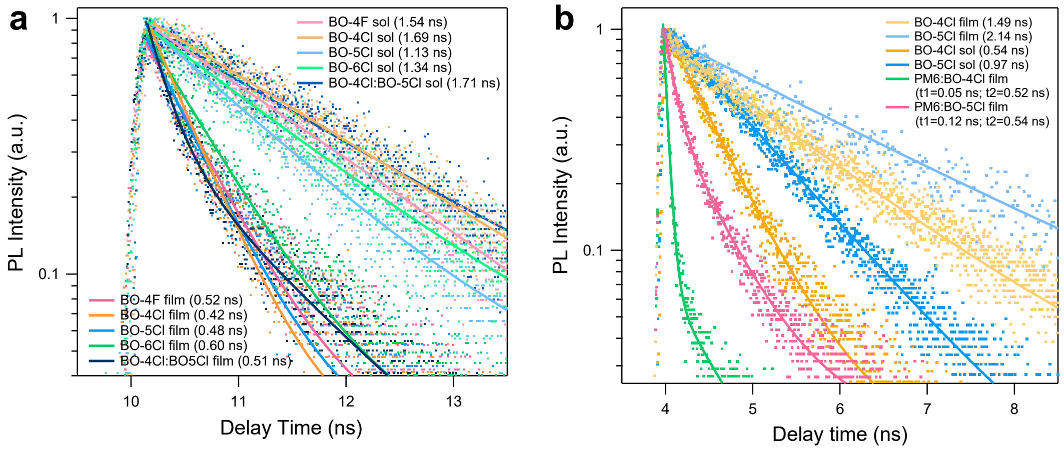

Supplementary Fig. 18 | a Normalized TRPL spectra of the acceptors in $CHCl_3$ solution and in films. **b** Normalized TRPL spectra of the acceptors in toluene at low concentration (0.05 mg mL^{-1}) and films blended with PS for the mass ratio of 1:50.

Quantum-chemical Calculations

All density functional theory (DFT) calculations were performed with the Gaussian 09 package.[3] Unless otherwise specified, geometry optimizations were carried out at the ωB97XD/6-31G(d,p) level of theory. All the electronic properties were calculated at the PCM-tuned-ωB97XD/6-31G(d,p) level of theory (*i.e.*, the range-separation parameter, ω, was optimized within the polarizable continuum model (PCM)[4] and using the so called gap-tuning procedure[5]; a typical dielectric constant of ε = 3.5 was used. For the PM6:BO-4F, PM6:BO-4Cl, PM6:BO-5Cl, and PM6:BO-6Cl complexes the optimally tuned ω values are obtained as 0.0085, 0.0080, 0.0092, and 0.0092 $bohr^{-1}$, respectively; while for the BO-4Cl and BO-5Cl dimers the tuned ω values are found as 0.0050 and 0.0057 $bohr^{-1}$, respectively. The lowest singlet excited states (*i.e.*, S_1) of the BO-4Cl and BO-5Cl molecules were examined by means of time-dependent DFT (TD-DFT) calculations. Based on the optimized geometries, the S_1 energies were calculated to be 1.83 and 1.91 eV for the BO-4Cl and BO-5Cl cases, respectively. The natural transition orbitals (NTOs) of the corresponding S_1 states are shown in **Supplementary Fig. 19**. The dipole moments for the ground states (S_0) and S_1 states were also evaluated. For the BO-4Cl and BO-5Cl cases, the dipole moments for the S_0 [S_1] states are 0.62 [4.36] and 3.30 [15.06] Debye, respectively. The dipole moments of BO-4Cl in both S_0 and S_1 states are oriented along the short molecular axis, while for BO-5Cl they are oriented predominantly along the long molecular axis. The relaxation energies related to the reduction (*i.e.*, addition of an electron) of the acceptor molecules were estimated to be 99, 90, 91, and 96 meV for the BO-4F, BO-4Cl, BO-5Cl, and BO-6Cl cases, respectively; the geometry optimizations were carried out at the PCM-tuned-ωB97XD/6-31G(d,p) level of theory.

At the computational level, the heterojunction interface structures are usually produced by the classical molecular dynamics (MD) simulations; based on the MD

outputs, the donor:acceptor complexes are extracted for the following investigation of the electronic properties. While such MD simulations for PM6:BO-4F, PM6:BO-4Cl, PM6:BO-5Cl, and PM6:BO-6Cl blends are under consideration, here we turn to the model interfacial donor:acceptor structures obtained by conventional geometry optimizations. We constructed donor:acceptor complexes by placing the end groups of the acceptors on top of the electron poor unit (BDD) or electron rich unit (BDT) of the PM6 donor (a PM6 dimer); the initial distance along the π-π interaction direction was set to ~3.5 Å. For the symmetric BO-4F and BO-4Cl acceptors, two donor:acceptor complexes were produced (*i.e.*, the end group on top of BDD or BDT), while for the asymmetric BO-5Cl and BO-6Cl acceptors, four donor:acceptor complexes were produced (*i.e.*, one end group on top of BDD or BDT, the other end group on top of BDD or BDT). Then, the geometries of all the donor:acceptor complexes were fully optimized (all the atoms in both acceptor and donor were allowed to move), and the lowest singlet LE and CT excited states were examined by means of time-dependent DFT calculations.The transfer integrals relevant for the electron-transport process were derived for the BO-4Cl and BO-5Cl crystals, according to the following procedure: (1) all different molecular dimers (*i.e.*, two acceptor molecules directly interacting with each other through π-π interactions) were extracted from the BO-4Cl and BO-5Cl crystals (in total, three and four different dimers can be extracted, respectively); (2) the alkyl side chains were replaced by methyl groups and the dimer geometries were kept as those found in the crystals; (3) the transfer integrals for electron transfer were then evaluated were evaluated with a fragment orbital approach in combination with a basis set orthogonalization procedure.[6] The results are shown in **Supplementary Fig. 20**.

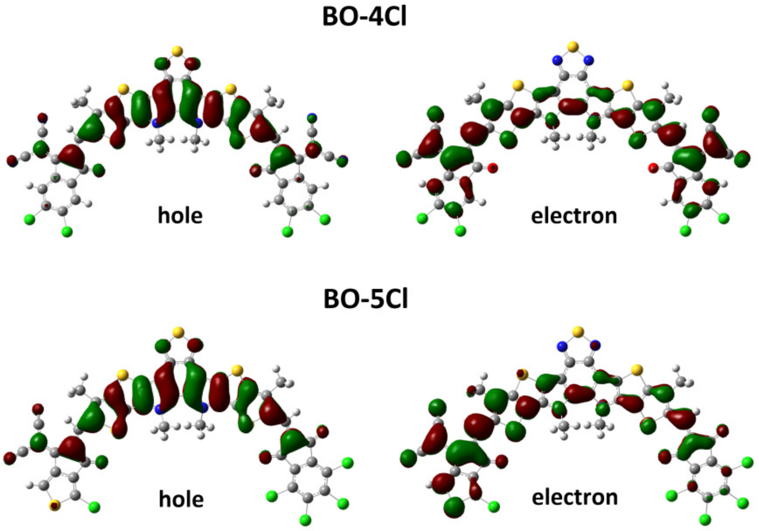

Supplementary Fig. 19 | Natural transition orbitals in the S_1 state of the BO-4Cl molecule and BO-5Cl molecule.

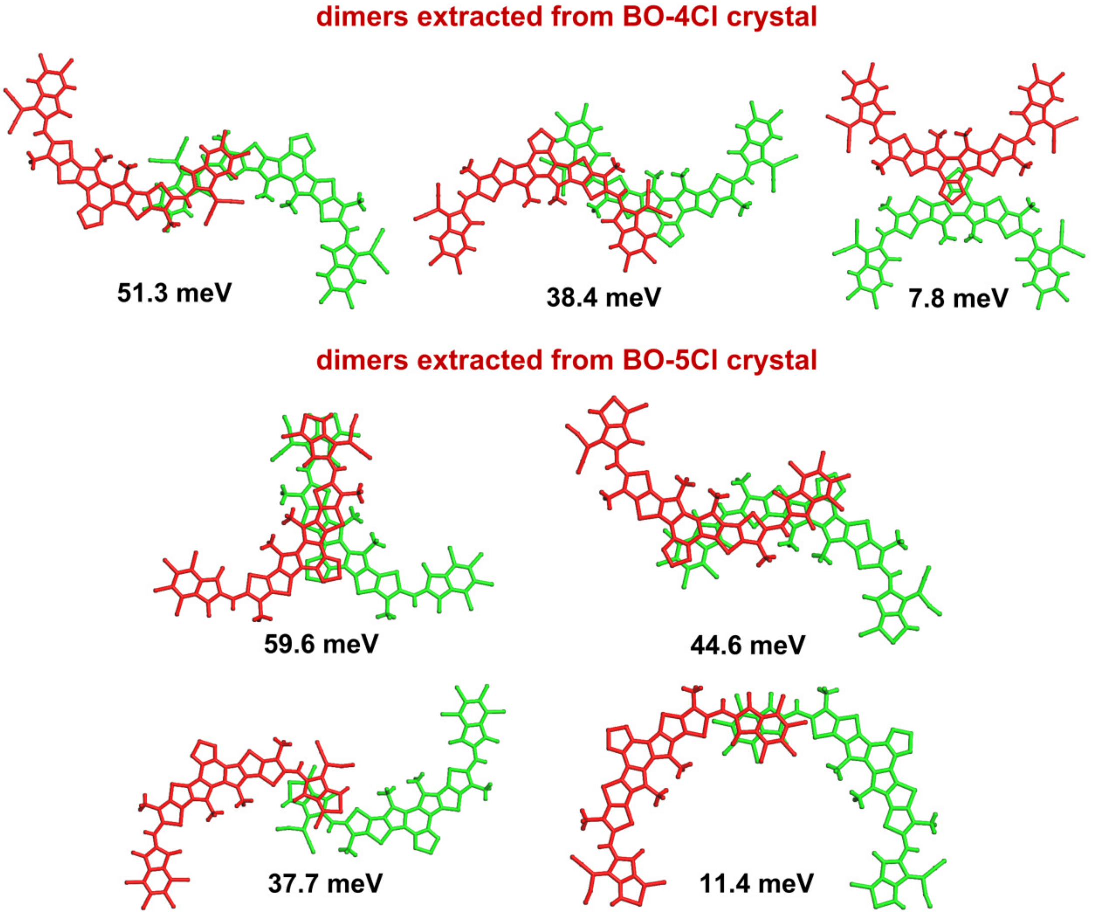

Supplementary Fig. 20 | Dimers extracted from the BO-4Cl and BO-5Cl crystals and corresponding transfer integrals (electronic couplings) related to the electron-transport process.

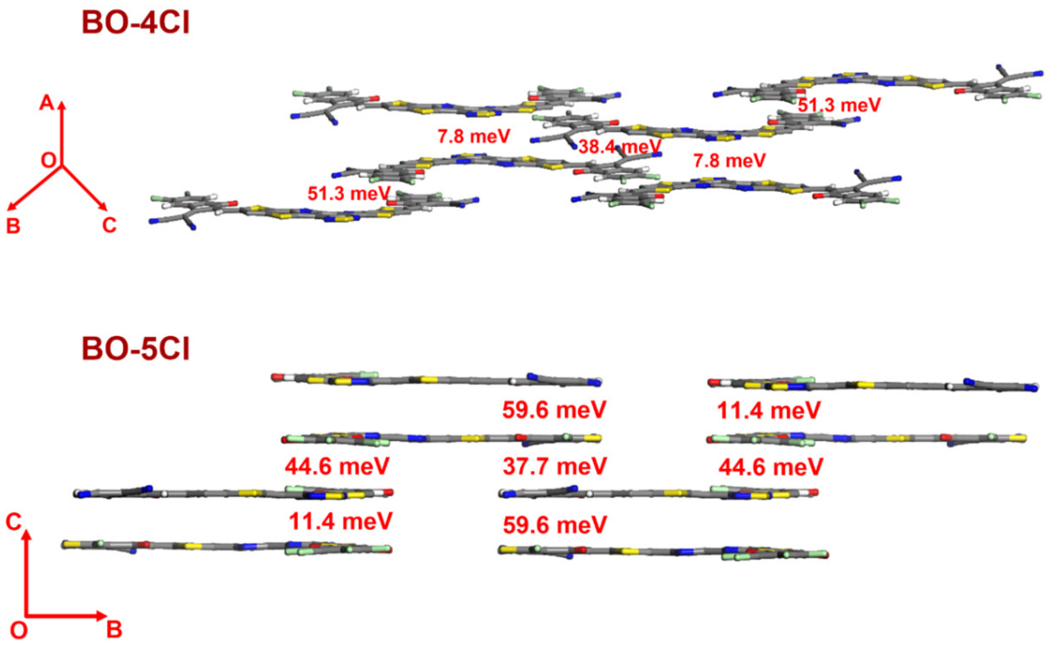

Supplementary Fig. 21 | Representative electron-transport pathways (formed by interacting molecules) in the BO-4Cl and BO-5Cl crystals.

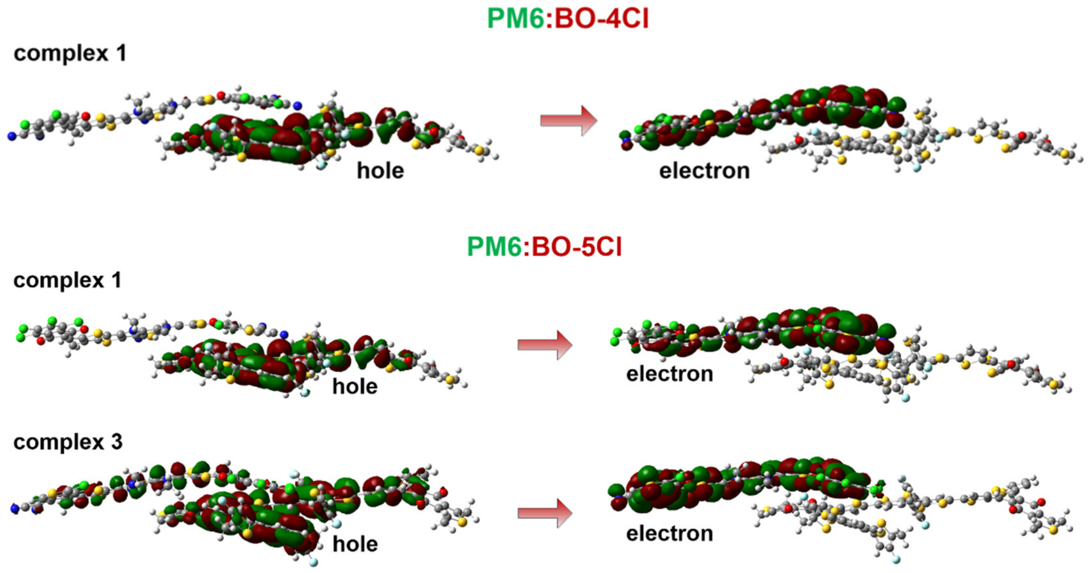

Supplementary Fig. 22 | NTOs for the CT states in PM6:BO-4Cl complex 1 as well as PM6:BO-5Cl complexes 1 and 3.

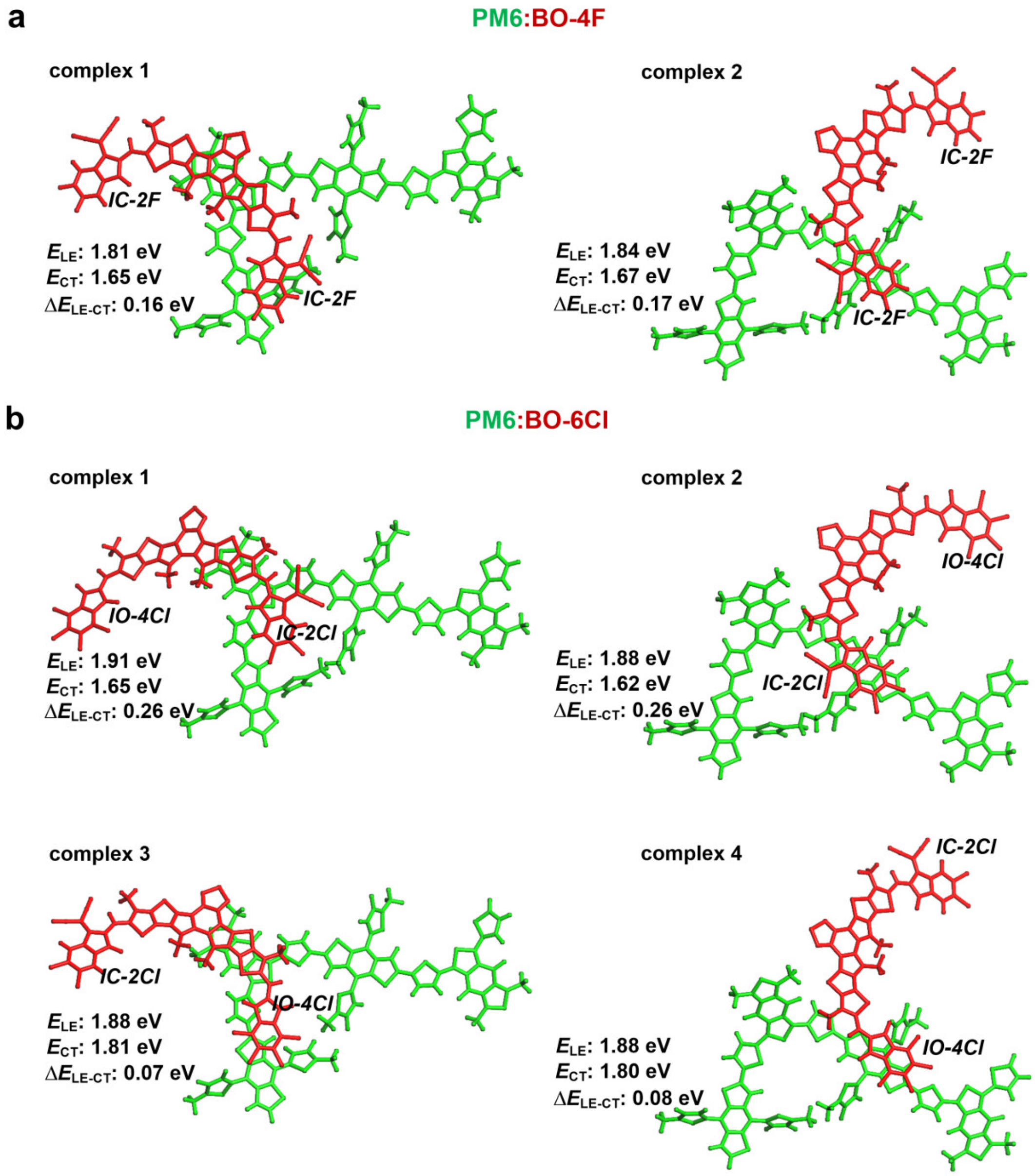

Supplementary Fig. 23 | Optimized **a** PM6:BO-4F and **b** PM6:BO-6Cl complexes as well as energies of their LE and CT states and their differences. The acceptor and PM6 donor are highlighted in red and green, respectively.

Energy Loss Calculation

The total energy loss (E_{loss}) can be attributed to three components following the equations below:

$$
\begin{aligned}
E_{\text{loss}} &= E_g - qV_{oc} \\
&= (E_g - qV_{oc}^{SQ}) + (qV_{oc}^{SQ} - qV_{oc}^{rad}) + (qV_{oc}^{rad} - qV_{oc}) \\
&= (E_g - qV_{oc}^{SQ}) + q\Delta V_{oc}^{rad,below\ gap} + q\Delta V_{oc}^{non-rad} \qquad (3) \\
&= \Delta E_1 + \Delta E_2 + \Delta E_3
\end{aligned}
$$

where E_g is the bandgap, q is the elementary charge, $\boldsymbol{V_{oc}^{SQ}}$ is the maximum voltage based on the Shockley-Queisser limit (SQ limit), $\boldsymbol{V_{oc}^{rad}}$ is the open-circuit voltage when there is only radiative recombination, $\Delta\boldsymbol{V_{oc}^{rad,below\ gap}}$ is the voltage loss of radiative recombination from the absorption below the bandgap and $\Delta\boldsymbol{V_{oc}^{non-rad}}$ is the voltage loss of non-radiative recombination.

ΔE_1 is due to radiative recombination from absorption above the bandgap, ΔE_2 is due to radiative recombination from absorption below the bandgap, and ΔE_3 is due to non-radiative recombination. The third component, the non-radiative loss, could be directly calculated by the following relation:

$$\Delta E_3 = q\Delta V_{oc}^{non-rad} = -kT\ln(EQE_{EL}) \qquad (4)$$

where k is the Boltzmann constant, T is temperature and EQE_{EL} is the radiative quantum efficiency of the OSCs when charge carriers are injected into the device in the dark.

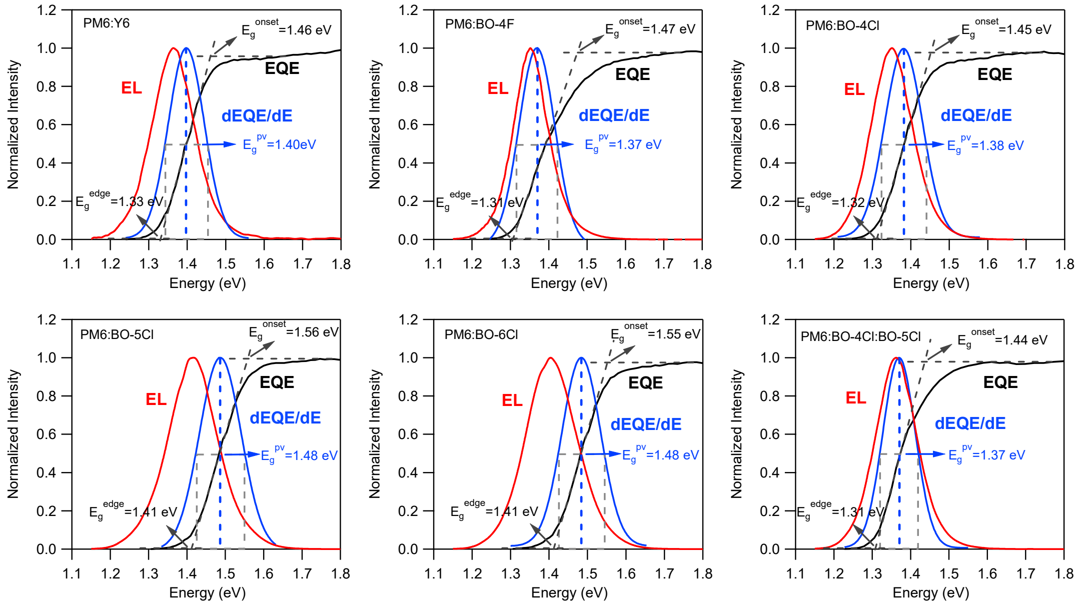

Supplementary Fig. 24 | Calculation of E_g with different methods; the one developed by Rau[7] is used in this work (blue line).

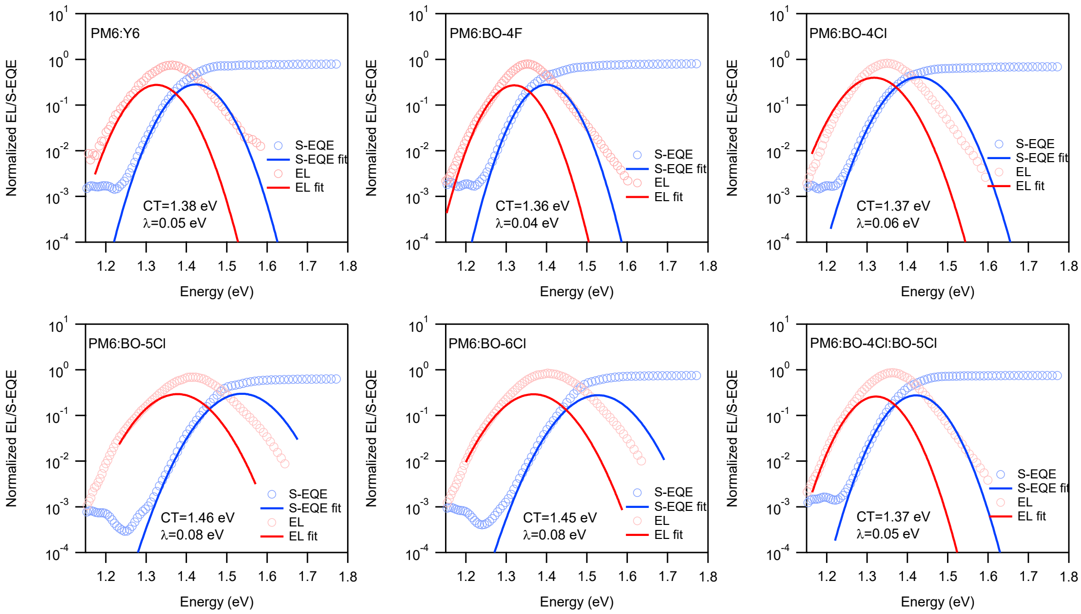

Supplementary Fig. 25 | The s-EQE and EL curves of the OSCs.

Certification Test Reports

Appendix: Summary of the Report
Report No.: GXgf2021-11241
Client:Zhejiang University
Sample: Organic Solar Cells
Type/Model: Organic Solar Cells
DUT S/N: HCL#-3-M1-F-1
Manufacturer: Zhejiang University
Date of Test: 05/13/2021
Temperature Sensor/Control System: None
Environmental conditions: (23.9±1) C°, RH (48.1±2) %
Mask: An aperture area of 4.728 mm^2 (M1, Certificate No.: CDjc2021-12227)

The test has been conducted by the PV Metrology Lab of NIM (National Institute of Metrology, China). Measurement of irradiance intensity and all other measurements are traceable to the International System of Units (SI). Data in this report apply only at the time of the test for the sample. For more details, please refer to the text of the report.

Forward Scan：

Area (mm^2)	I_{sc}(mA)	V_{oc}(V)	P_{max}(mW)
4.728	1.271	0.865	0.860
I_{max}(mA)	V_{max}(V)	FF (%)	η (%)
1.161	0.740	78.2	18.2

I-V Characterization Methods:
JJF 1622-2017: Calibration Specification of Solar Cells：Photoelectric Properties
Secondary Reference Cell:
Device S/N: 81#; Device Material: Mono-Si
Solar Simulator:
Classification: AAA (Double-light source: Xeon and Halogen);
Total irradiance: 1000 W/m^2 based on I_{sc} of the above Secondary Reference Cell.

Report No.: GXgf2021-11241

DUT S/N：HCL#-3-M1-F-1

Forward Scan:

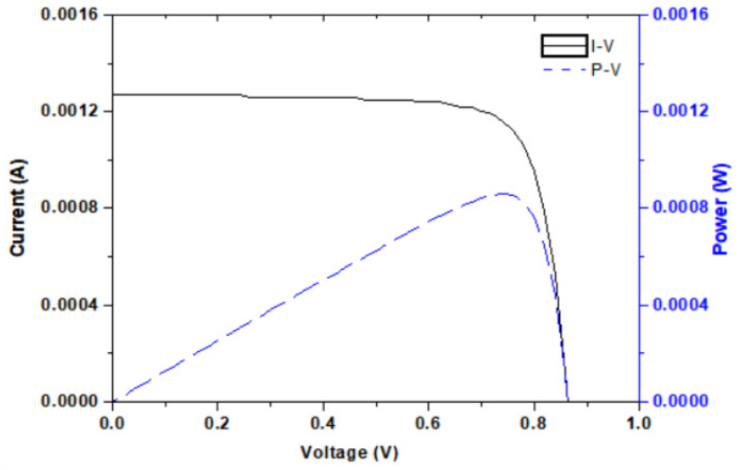

SCLC and Charge Recombination

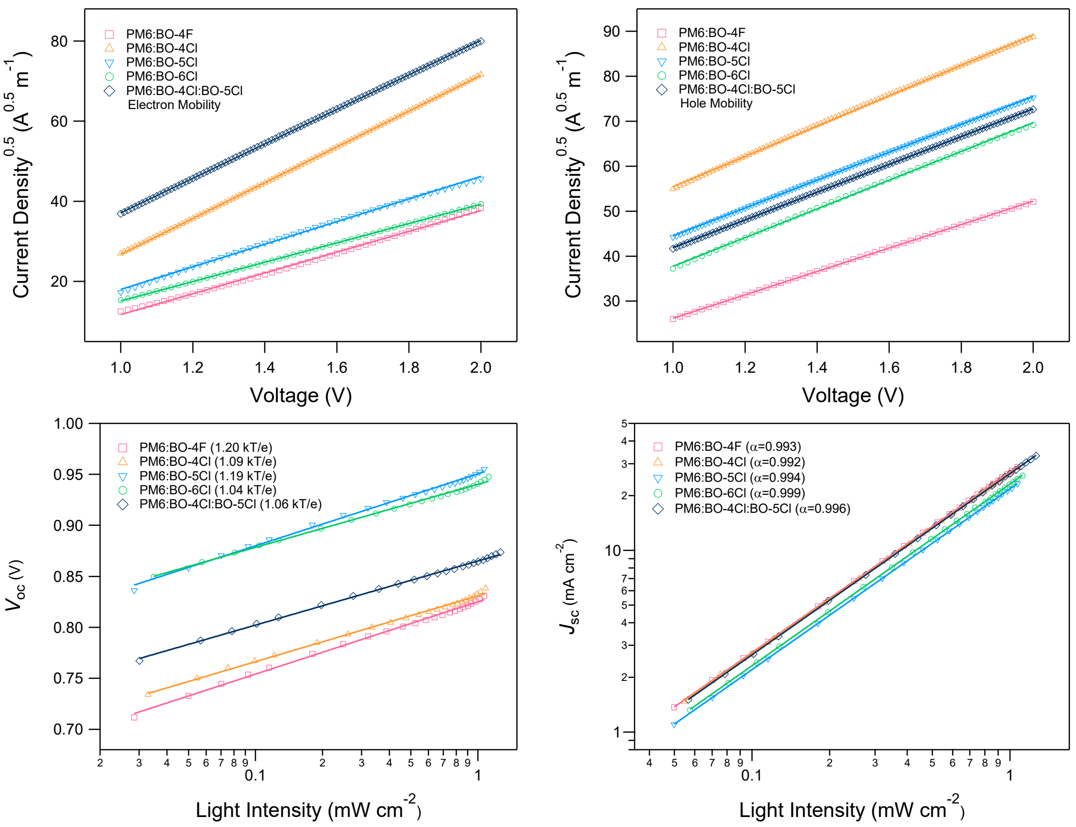

Supplementary Fig. 26 | a $J^{0.5}$-V curves of electron-only devices. **b** $J^{0.5}$-V curves of hole-only devices. **c** Dependence of V_{oc} on light intensity in the optimized devices. **d** Dependence of J_{sc} on light intensity in the optimized devices.

The charge carrier mobility of the donor:acceptor films were measured using the space charge limited current (SCLC) method. Electron-only devices were fabricated with the structure: ITO/ZnO/Active Layer/PFN-Br/Al, while hole-only devices used the structure: ITO/PEDOT:PSS/Active Layer/MoO_3/Ag. The device characteristics were extracted by modeling the dark current under forward bias via the SCLC expression described by the Mott-Gurney Law:

$$J=\frac{9}{8}\varepsilon_r\varepsilon_0\mu\frac{V^2}{L^3} \quad (5)$$

Here, $\varepsilon_r \approx 3.5$ is the average dielectric constant of the blend film, ε_0 is the permittivity of the free space, μ is the carrier mobility, $L \approx 100$ nm is the thickness of the film, and V is the applied voltage.

Supplementary Tables

Supplementary Table 1 Device parameters of reported OPVs based on Y-series asymmetric acceptors.

D:A	V_{oc} (V)	J_{sc} (mA cm^{-2})	FF	PCE (%)	Ref.
PM6:BP5T-4F	0.888	24.6	76.3	16.7	8
PM6:BP4T-4F	0.839	26.3	77.7	17.1	
PM6:ABP4T-4F	0.922	22.0	75.1	15.2	
PM6:Bu-OD-4F	0.85	26.2	76.6	17.10	9
PM6:BO-4F	0.84	27.0	76.7	17.39	
PM6:EH-HD-4F	0.84	27.5	79.3	18.38	
PM6:Y6-2O	0.92	13.3	53.5	6.6	10
PM6:Y6-1O	0.89	23.2	78.3	16.1	
PM6:Y6	0.82	25.3	75.5	15.7	
PM6:BTP-S1	0.934	22.39	72.69	15.21	2
PM6:BTP-S2	0.945	24.07	72.02	16.37	
PM6:Y6	0.842	26.05	72.03	15.79	
PM6:BTP-S8	0.852	26.96	75.45	17.33	11
PM6:BTP-S9	0.846	26.47	78.44	17.56	
PM6:BO-4Cl	0.845	26.70	75.83	17.21	
PM6:BTP-T-2Cl	0.936	22.32	71.59	14.89	12
PM6:BTP-T-3Cl	0.893	26.02	75.79	17.61	
PM6:BTP-T-4Cl-BO	0.844	26.74	76.19	17.20	

Supplementary Table 2 UV-vis and PL results for the pure acceptors in $CHCl_3$ solution and in films.

Material	UV-vis			PL	
	λ_{max}^{sol} (nm)	λ_{max}^{film} (nm)	λ_{edge}^{film} (nm)	λ_{max}^{sol} (nm)	λ_{max}^{film} (nm)
BO-4F	728	830	924	781	913
BO-4Cl	742	840	935	795	912
BO-5Cl	712	778	874	776	874
BO-6Cl	711	782	884	783	879

Supplementary Table 3 PL results of the five blends.

Material	PL λ_{max}^{film} (nm)
PM6:BO-4F	909
PM6:BO-4Cl	909
PM6:BO-5Cl	871
PM6:BO-6Cl	872
PM6:BO-4Cl:BO-5Cl	900

Supplementary Table 4 CV and UPS results.

Material	$E_{HOMO/LUMO}$ (eV)	E_g^{cv} (eV)	ΔHOMO (eV)
BO-4F	-5.69/-3.94	1.75	0.076
BO-4Cl	-5.68/-3.94	1.74	0.070
BO-5Cl	-5.62/-3.86	1.76	0.026
BO-6Cl	-5.63/-3.88	1.75	0.035

[a] HOMO differences between PM6 and acceptors, which were calculated from the UPS results.

Supplementary Table 5 PL results of the pure acceptors (at low concentration (0.05 mg mL^{-1}) in toluene & blended with PS for the mass ratio of 1:50).

Material	PL	
	λ_{max}^{sol} (nm)	λ_{max}^{film} (nm)
BO-4Cl	770	889
BO-5Cl	740	856

Supplementary Table 6 TRPL results of the acceptors in $CHCl_3$ solution and films.

Material	TRPL (ns)	
	sol	film
BO-4F	1.54	0.52
BO-4Cl	1.69	0.42
BO-5Cl	1.13	0.48
BO-6Cl	1.34	0.60
BO-4Cl:BO-5Cl	1.71	0.51

Supplementary Table 7 TRPL results of the pure acceptors (at low concentration (0.05 mg mL^{-1}) in toluene & blended with PS in the mass ratio of 1:50) and their blends.

Material	TRPL (ns)		
	sol	film	blend
BO-4Cl	0.54	1.49	0.05
BO-5Cl	0.97	2.14	0.12

Supplementary Table 8 Relaxation energies.

Material	λ (eV)
BO-4F	0.099
BO-4Cl	0.090
BO-5Cl	0.091
BO-6Cl	0.096

Supplementary Table 9 Device fabrication conditions.

Active Layer	D:A (wt. ratio)	Additive	Concentration	Annealing
PM6:Y6	1:1.2	0.5% CN	17.6 mg mL^{-1}	100°C 10min
PM6:BO-4F	1:1.2	0.5% CN	17.6 mg mL^{-1}	100°C 10min
PM6:BO-4Cl	1:1.2	0.5% DIO	17.6 mg mL^{-1}	100°C 10min
PM6:BO-5Cl	1:1.2	0.5% CN	17.6 mg mL^{-1}	120°C 10min
PM6:BO-6Cl	1:1.2	0.5% CN	17.6 mg mL^{-1}	120°C 10min
PM6:BO-4Cl:BO-5Cl	1:0.96:0.24	0.3% DIO	17.6 mg mL^{-1}	80°C 10min

Supplementary Table 10 Device parameters of PM6:BO-5Cl under different optimization conditions.

D:A (wt. ratio)	Annealing	Additive	V_{oc} (V)	J_{sc} (mA cm^{-2})	FF	PCE (%)
1:1	100°C	/	0.950	21.21	0.614	12.41 (12.33±0.11)
1:1.2	100°C	/	0.949	20.65	0.654	12.86 (12.73±0.13)
1:1.5	100°C	/	0.950	20.72	0.637	12.50 (12.30±0.26)
1:1.2	100°C	0.5% DIO	0.934	22.30	0.648	13.45 (13.32±0.12)
1:1.2	100°C	0.5% CN	0.960	22.19	0.695	14.74 (14.66±0.08)
1:1.2	100°C	0.3% CN	0.960	21.75	0.690	14.34 (14.19±0.15)
1:1.2	100°C	1.0% CN	0.959	21.97	0.694	14.57 (14.46±0.08)
1:1.2	120°C	0.5% CN	0.958	22.57	0.701	15.02 (14.88±0.07)
1:1.2	80°C	0.5% CN	0.956	22.07	0.696	14.63 (14.42±0.13)

Supplementary Table 11 Device parameters of PM6:BO-4Cl:BO-5Cl under different optimization conditions (Total D:A wt. ratio is kept as 1:1.2 and the percentage refers to the amount of BO-5Cl incorporated).

D:A (wt. ratio)	Annealing	Additive	V_{oc} (V)	J_{sc} (mA cm^{-2})	FF	PCE (%)
1:1.2 (10%)	100°C	/	0.865	26.29	0.765	17.44 (17.36±0.09)
1:1.2 (20%)	100°C	/	0.876	26.34	0.768	17.69 (17.57±0.07)
1:1.2 (30%)	100°C	/	0.881	25.78	0.765	17.42 (17.23±0.13)
1:1.2 (50%)	100°C	/	0.890	26.36	0.726	16.94 (16.65±0.24)
1:1.2 (20%)	100°C	0.5% CN	0.878	26.47	0.771	17.93 (17.75±0.15)
1:1.2 (20%)	100°C	0.5% DIO	0.869	26.51	0.783	18.09 (17.99±0.11)
1:1.2 (20%)	100°C	1.0% DIO	0.864	26.40	0.773	17.65 (17.54±0.12)
1:1.2 (20%)	100°C	0.3% DIO	0.872	26.94	0.779	18.31 (18.20±0.12)
1:1.2 (20%)	120°C	0.3% DIO	0.868	26.54	0.784	18.08 (17.95±0.09)
1:1.2 (20%)	80°C	0.3% DIO	0.874	26.93	0.788	18.56 (18.24±0.16)

Supplementary Table 12 SCLC results.

Active Layer	Mobility ($cm^2\ V^{-1}\ s^{-1}$)		
	Electron (μ_e)	Hole (μ_h)	μ_h/μ_e
PM6:BO-4F	1.88×10^{-4}	1.95×10^{-4}	1.04
PM6:BO-4Cl	5.53×10^{-4}	3.27×10^{-4}	0.59
PM6:BO-5Cl	2.45×10^{-4}	2.75×10^{-4}	1.12
PM6:BO-6Cl	1.74×10^{-4}	2.86×10^{-4}	1.64
PM6:BO-4Cl:BO-5Cl	5.26×10^{-4}	2.71×10^{-4}	0.51

Supplementary Table 13 Detailed parameters of GIWAXS profiles along with in-plane direction.

Active Layer	Position/Å^{-1}	FWHM	*d*-spacing/ Å	CL/ Å
PM6	0.292	0.084	21.5	67.3
BO-4F	0.312	0.114	20.1	49.6
	0.389	0.061	16.1	93.4
	0.458	0.107	13.7	52.6
BO-4Cl	0.337	0.136	18.6	41.6
	0.389	0.058	16.2	97.3
	0.443	0.131	14.2	43.3
BO-5Cl	0.230	0.028	27.3	204
	0.309	0.139	20.3	40.7
	0.416	0.145	15.1	39.0
BO-6Cl	0.357	0.165	17.6	34.3
	0.384	0.073	16.4	77.9
PM6: BO-4F	0.298	0.068	21.1	82.6
	0.379	0.084	16.6	67.0
PM6: BO-4Cl	0.301	0.077	20.9	73.5
	0.386	0.081	16.3	69.8
PM6: BO-5Cl	0.296	0.060	21.2	94.8
	0.323	0.167	19.4	33.8
PM6: BO-6Cl	0.300	0.073	21.0	77.6
	0.375	0.101	16.7	55.7
PM6: BO-4Cl:BO-5Cl	0.301	0.069	20.9	81.8
	0.376	0.065	16.7	87.1

Supplementary Table 14 Detailed parameters of GIWAXS profiles along with out-of-plane direction.

Active Layer	Position/Å^{-1}	FWHM	*d*-spacing/ Å	CL/ Å
PM6	1.69	0.267	3.72	21.1
BO-4F	1.77	0.196	3.54	28.9
BO-4Cl	1.77	0.174	3.55	32.5
BO-5Cl	1.77	0.189	3.56	29.9
BO-6Cl	1.76	0.189	3.56	30.0
PM6: BO-4F	1.74	0.219	3.60	25.8
PM6: BO-4Cl	1.74	0.212	3.61	26.7
PM6: BO-5Cl	1.73	0.213	3.63	26.5
PM6: BO-6Cl	1.74	0.212	3.61	26.6
PM6: BO-4Cl:BO-5Cl	1.74	0.219	3.62	25.8

Supplementary Table 15 Morphology parameters fitted by the GISAXS profiles (ξ is the intermixing domain size; 2R_g is the crystal domain size).

Active Layer	ξ (nm)	2R_g (nm)
PM6:BO-4F	29.9	9.4
PM6:BO-4Cl	49.3	10.9
PM6:BO-5Cl	23.2	12.1
PM6:BO-6Cl	60.1	12.0
PM6:BO-4Cl:BO-5Cl	29.8	11.2

Supplementary Table 16 Comparison of efficiency and energy loss between this work and earlier references.

Active Layer	E_g	V_{oc}	E_{loss}	ΔE_3	PCE (%)	Ref.
PM6:BO-4Cl:BO5Cl	1.37	0.872	0.498	0.198	18.36	this work
PM6:BO5Cl	1.48	0.958	0.522	0.178	15.02	this work
PM6:BTP-4F-12	1.4	0.85	0.55	/	16.4	1
PM6:BTP-S1	1.49	0.93	0.56	0.22	15.21	2
PM6:BTP-S2	1.48	0.95	0.53	0.2	16.37	2
PM6:BP4T-4F	1.389	0.839	0.55	0.288	17.1	8
PM6:BP5T-4F	1.418	0.888	0.53	0.242	16.7	8
PM6:ABP4T-4F	1.454	0.922	0.531	0.206	15.2	8
PNTB-2T:Y6	1.407	0.872	0.535	0.222	16.72	13
PNTB-2T:Y6:PC71BM	1.407	0.875	0.533	0.217	17.35	13
PM6:BTP-4F-12:MeIC	1.389	0.863	0.526	0.227	17.4	14
PFBCPZ:IT-4F	1.57	0.92	0.65	0.29	15.3	15
PBDB-T:Y1	1.44	0.87	0.57	0.25	13.42	16
PBDB-T:Y2	1.4	0.82	0.58	0.26	13.4	16
PM6:Y6	1.4	0.83	0.57	0.23	15.6	17
PM6:BTP-4Cl	1.4	0.87	0.53	0.21	16.5	17
PBDB-T:PYTT-2	1.52	0.91	0.61	0.19	14.32	18

PBDB-T:IT4F:NMR-1	1.53	0.77	0.76	0.36	11.47	19
PM6:ITCPTC	1.65	0.95	0.7	0.36	12.3	20
PM6:ITC-2Cl	1.58	0.91	0.67	0.32	13.6	20
PM6:IT-4F	1.6	0.87	0.73	0.37	12.9	20
PM6:IT-4Cl	1.56	0.8	0.76	0.41	12.7	20
PBDB-T:FOIC	1.391	0.751	0.751	0.318	11.86	21
PTB7-Th:IOTIC-4F	1.34	0.716	0.62	0.338	10.2	22
PTB7-Th:IOTIC-2F	1.36	0.792	0.568	0.305	7.2	22
PTB7-Th:IOTIC	1.44	0.88	0.56	0.29	6	22
PBDB-T:IEICO-4F	1.35	0.75	0.6	0.28	6.64	23
P2F-Ehp:IT-2F	1.63	0.89	0.74	0.33	12.96	24
BTR:NITI:PC71BM	1.49	0.94	0.55	0.3	13.63	25
PFBDB-T:C8-ITIC	1.53	0.93	0.6	0.33	13.2	26
PBDB-T:H1	1.41	0.76	0.66	0.33	14.06	27
PBDB-T:H2	1.42	0.79	0.63	0.31	15.12	27
PffBT2T-TT:O-IDTBR	1.6	1.05	0.55	0.24	10.4	28
S1:Y6	1.41	0.87	0.54	0.23	16.42	29
PBDB-T:o-4TBC-2F	1.39	0.76	0.63	0.3	10.26	30
PM6:Y6:S3	1.435	0.856	0.579	0.223	17.53	31
S3:Y6	1.432	0.89	0.542	0.193	16.12	31
J71:ITC6-IC	1.67	0.95	0.72	0.37	10.41	32
PTQ10:Y6	1.42	0.87	0.55	0.23	16.21	33
PM6:Y6-Se	/	0.82	0.55	0.22	15.82	34
PM6:Y6-2Se	/	0.83	0.56	0.24	14.62	34
D18:Y6Se	/	0.839		0.215	17.7	35
PTVT-T: eC9	/	0.79	0.61	0.241	16.2	36
L2:TTPT-T-4F	1.58	0.86	0.72	0.33	14	37
PTB7-Th:PBDTm-T1:FOIC	1.38	0.76	0.62	0.27	13.8	38
PE71:Y6	1.37	0.82	0.545	0.24	12.03	39
PE72:Y6	1.37	0.83	0.535	0.23	9.74	39
PM6:Y6:C8-DTC	1.41	0.873	0.537	0.215	17.52	40
PBDB-T:H3	1.31	0.76	0.55	0.25	13.75	41
PBDB-T:DOC2C6-2F	1.42	0.85	0.57	0.27	13.24	42
PM6:Y6:3TP3T-4F	1.41	0.85	0.56	0.22	16.7	43
ZR1:Y6	1.4	0.86	0.54	0.24	14.34	44
PM6:NOIC	1.62	0.89	0.73	0.28	11.4	45
PM6:NOIC1	1.46	0.86	0.6	0.22	12.5	45
PM6:NOIC2	1.57	0.93	0.64	0.28	14.1	45
PM6:NOIC4	1.62	0.94	0.68	0.25	10.1	45

Supplementary References

1 Hong, L. *et al.* Eco-Compatible Solvent-Processed Organic Photovoltaic Cells with Over 16% Efficiency. *Adv. Mater.* **31**, 1903441 (2019).

2 Li, S. *et al.* Asymmetric Electron Acceptors for High-Efficiency and Low-Energy-Loss Organic Photovoltaics. *Adv. Mater.* **32**, 2001160 (2020).

3 Kyaw, A. K. *et al.* Improved light harvesting and improved efficiency by insertion of an optical spacer (ZnO) in solution-processed small-molecule solar cells. *Nano Lett.* **13**, 3796-3801 (2013).

4 Mennucci, B. & Tomasi, J. Continuum solvation models: A new approach to the problem of solute's charge distribution and cavity boundaries. *J. Chem. Phys.* **106**, 5151-5158 (1997).

5 Kronik, L., Stein, T., Refaely-Abramson, S. & Baer, R. Excitation Gaps of Finite-Sized Systems from Optimally Tuned Range-Separated Hybrid Functionals. *J. Chem. Theory. Comput.* **8**, 1515-1531 (2012).

6 Coropceanu, V. *et al.* Charge Transport in Organic Semiconductors. *Chem. Rev.* **107**, 926-952 (2007).

7 Rau, U., Blank, B., Müller, T. C. M. & Kirchartz, T. Efficiency Potential of Photovoltaic Materials and Devices Unveiled by Detailed-Balance Analysis. *Phys. Rev. Appl.* **7**, 044016 (2017).

8 Gao, W. *et al.* Asymmetric Acceptors Enabling Organic Solar Cells to Achieve an over 17% Efficiency: Conformation Effects on Regulating Molecular Properties and Suppressing Nonradiative Energy Loss. *Adv. Energy Mater.* **11**, 2003177 (2020).

9 Chen, S. *et al.* High-performance polymer solar cells with efficiency over 18% enabled by asymmetric side chain engineering of non-fullerene acceptors. *Sci. China Chem.* **64**, 1192-1199 (2021).

10 Chen, Y. *et al.* Asymmetric Alkoxy and Alkyl Substitution on Nonfullerene Acceptors Enabling High-Performance Organic Solar Cells. *Adv. Energy Mater.* **11**, 2003141 (2020).

11 Li, S. *et al.* Unveiling structure-performance relationships from multi-scales in non-fullerene organic photovoltaics. *Nat. Commun.* **12**, 4627 (2021).

12 Pan, Y. *et al.* A New End Group on Nonfullerene Acceptors Endows Efficient Organic Solar Cells with Low Energy Losses. *Adv. Funct. Mater.* **32**, 2108614

(2021).

13 Zhang, G. *et al.* Naphthalenothiophene imide-based polymer exhibiting over 17% efficiency. *Joule* **5**, 931-944 (2021).

14 Ma, X. *et al.* Achieving 17.4% Efficiency of Ternary Organic Photovoltaics with Two Well-Compatible Nonfullerene Acceptors for Minimizing Energy Loss. *Adv. Energy Mater.* **10**, 2001404 (2020).

15 Wu, J. *et al.* Carboxylate substituted pyrazine: A simple and low-cost building block for novel wide bandgap polymer donor enables 15.3% efficiency in organic solar cells. *Nano Energy* **82**, 105679 (2021).

16 Yuan, J. *et al.* Enabling low voltage losses and high photocurrent in fullerene-free organic photovoltaics. *Nat. Commun.* **10**, 570, (2019).

17 Cui, Y. *et al.* Over 16% efficiency organic photovoltaic cells enabled by a chlorinated acceptor with increased open-circuit voltages. *Nat. Commun.* **10**, 2515 (2019).

18 Wang, T. *et al.* Highly Efficient and Stable All-Polymer Solar Cells Enabled by Near-Infrared Isomerized Polymer Acceptors. *Chem. Mater.* **33**, 761-773 (2021).

19 Qin, Y. *et al.* Reduced Nonradiative Energy Loss Caused by Aggregation of Nonfullerene Acceptor in Organic Solar Cells. *Adv. Energy Mater.* **9**, 1901823 (2019).

20 Luo, Z. *et al.* Reduced Energy Loss Enabled by a Chlorinated Thiophene-Fused Ending-Group Small Molecular Acceptor for Efficient Nonfullerene Organic Solar Cells with 13.6% Efficiency. *Adv. Energy Mater.* **9**, 1900041 (2019).

21 Wang, Y. *et al.* Achieving Balanced Crystallization Kinetics of Donor and Acceptor by Sequential-Blade Coated Double Bulk Heterojunction Organic Solar Cells. *Adv. Energy Mater.* **10**, 2000826 (2020).

22 Karki, A. *et al.* Unifying Charge Generation, Recombination, and Extraction in Low-Offset Non-Fullerene Acceptor Organic Solar Cells. *Adv. Energy Mater.* **10**, 2001203 (2020).

23 Zhan, L. *et al.* A Near-Infrared Photoactive Morphology Modifier Leads to Significant Current Improvement and Energy Loss Mitigation for Ternary

Organic Solar Cells. *Adv. Sci.* **5**, 1800755 (2018).

24 Fan, B. *et al.* Fine-tuning of the chemical structure of photoactive materials for highly efficient organic photovoltaics. *Nat. Energy* **3**, 1051-1058 (2018).

25 Zhou, Z. *et al.* High-efficiency small-molecule ternary solar cells with a hierarchical morphology enabled by synergizing fullerene and non-fullerene acceptors. *Nat. Energy* **3**, 952-959 (2018).

26 Fei, Z. *et al.* An Alkylated Indacenodithieno[3,2-b]thiophene-Based Nonfullerene Acceptor with High Crystallinity Exhibiting Single Junction Solar Cell Efficiencies Greater than 13% with Low Voltage Losses. *Adv. Mater.* **30**, 1705209 (2018).

27 Qin, L. *et al.* Triplet Acceptors with a D-A Structure and Twisted Conformation for Efficient Organic Solar Cells. *Angew. Chem. Int. Ed.* **59**, 15043-15049 (2020).

28 Chen, S. *et al.* Efficient Nonfullerene Organic Solar Cells with Small Driving Forces for Both Hole and Electron Transfer. *Adv. Mater.* **30**, 1804215 (2018).

29 Sun, H. *et al.* A monothiophene unit incorporating both fluoro and ester substitution enabling high-performance donor polymers for non-fullerene solar cells with 16.4% efficiency. *Energy Environ. Sci.* **12**, 3328-3337 (2019).

30 Chen, Y.-N. *et al.* A Fully Non-fused Ring Acceptor with Planar Backbone and Near-IR Absorption for High Performance Polymer Solar Cells. *Angew. Chem. Int. Ed.* **59**, 22714-22720 (2020).

31 An, Q. *et al.* Two compatible polymer donors contribute synergistically for ternary organic solar cells with 17.53% efficiency. *Energy Environ. Sci.* **13**, 5039-5047 (2020).

32 Sun, R. *et al.* A multi-objective optimization-based layer-by-layer blade-coating approach for organic solar cells: rational control of vertical stratification for high performance. *Energy Environ. Sci.* **12**, 3118-3132 (2019).

33 Sun, C. *et al.* Achieving Fast Charge Separation and Low Nonradiative Recombination Loss by Rational Fluorination for High-Efficiency Polymer Solar Cells. *Adv. Mater.* **31**, 1905480 (2019).

34 Tong, Y. *et al.* Progress of the key materials for organic solar cells. *Sci. China Chem.* **63**, 758-765 (2020).

35 Zhang, Z. *et al.* Selenium Heterocyclic Electron Acceptor with Small Urbach Energy for As-Cast High-Performance Organic Solar Cells. *J. Am. Chem. Soc.* **142**, 18741-18745 (2020).

36 Ren, J. *et al.* Molecular design revitalizes the low-cost PTV-polymer for highly efficient organic solar cells. *Natl. Sci. Rev.* **8**, nwab031 (2021).

37 Li, X. *et al.* Non-Fullerene Organic Solar Cells Based on Benzo[1,2-b:4,5-b′]difuran-Conjugated Polymer with 14% Efficiency. *Adv. Funct. Mater.* **30**, 1906809 (2019).

38 Xie, Y. *et al.* Ternary Organic Solar Cells with Small Nonradiative Recombination Loss. *ACS Energy Lett.* **4**, 1196-1203 (2019).

39 Zhang, B. *et al.* The first application of isoindigo-based polymers in non-fullerene organic solar cells. *Sci. China Chem.* **63**, 1262–1271 (2020).

40 Li, D. *et al.* Non-fullerene acceptor fibrils enable efficient ternary organic solar cells with 16.6% efficiency. *Sci. China Chem.* **63**, 1461-1468 (2020).

41 He, C. *et al.* Near infrared electron acceptors with a photoresponse beyond 1000 nm for highly efficient organic solar cells. *J. Mater. Chem. A* **8**, 18154-18161 (2020).

42 Huang, H. *et al.* Noncovalently fused-ring electron acceptors with near-infrared absorption for high-performance organic solar cells. *Nat. Commun.* **10**, 3038 (2019).

43 Song, J. *et al.* Ternary Organic Solar Cells with Efficiency >16.5% Based on Two Compatible Nonfullerene Acceptors. *Adv. Mater.* **31**, 1905645 (2019).

44 Zhou, R. *et al.* All-small-molecule organic solar cells with over 14% efficiency by optimizing hierarchical morphologies. *Nat. Commun.* **10**, 5393 (2019).

45 Li, T. *et al.* Butterfly Effects Arising from Starting Materials in Fused-Ring Electron Acceptors. *J. Am. Chem. Soc.* **142**, 20124-20133 (2020).
