# Supplementary material for: Asymmetric electron acceptor enables highly luminescent organic solar cells with certified efficiency over 18%
Source: Nat Commun. 2022 May 11;13:2598. doi: 10.1038/s41467-022-30225-7 (PMC9095617; doi:10.1038/s41467-022-30225-7)
Supplement: Supplementary file 2 — Solar Cells Reporting Summary [file 41467_2022_30225_MOESM2_ESM.pdf]

nature research

Corresponding author(s): Hongzheng Chen

Solar Cells Reporting Summary

Nature Research wishes to improve the reproducibility of the work that we publish. This form is intended for publication with all accepted papers reporting the characterization of photovoltaic devices and provides structure for consistency and transparency in reporting. Some list items might not apply to an individual manuscript, but all fields must be completed for clarity.

For further information on Nature Research policies, including our data availability policy, see Authors & Referees.

Experimental design

Please check: are the following details reported in the manuscript?

1. Dimensions

Item	Yes/No	Response
Area of the tested solar cells	☒ Yes ☐ No	The device was completed with an active area of 0.0925 cm2, described in the section of "Methods" in the Manuscript. / *Explain why this information is not reported/not relevant.*
Method used to determine the device area	☒ Yes ☐ No	The device was completed with an active area of 0.0925 cm2, as defined by the overlapping area of ITO and Ag, described in the section of "Methods" in the Manuscript. / *Explain why this information is not reported/not relevant.*

2. Current-voltage characterization

Item	Yes/No	Response
Current density-voltage (J-V) plots in both forward and backward direction	☐ Yes ☒ No	*State where this information can be found in the text.* / Organic solar cells do not have hysteresis problems. The devices were scanned in forward direction.
Voltage scan conditions<br>*For instance: scan direction, speed, dwell times*	☒ Yes ☐ No	The scan direction is -0.2 V to 1.2 V, with a scan step of 0.01 V and dwell time is 1 ms, described in the section of "Methods" in the Manuscript. / *Explain why this information is not reported/not relevant.*
Test environment<br>*For instance: characterization temperature, in air or in glove box*	☒ Yes ☐ No	Devices were tested at room temperature (ca. 25 Celsius degree) in N2-filled glove box, described in the section of "Methods" in the Manuscript. / *Explain why this information is not reported/not relevant.*
Protocol for preconditioning of the device before its characterization	☐ Yes ☒ No	*State where this information can be found in the text.* / No preconditioning protocol.
Stability of the J-V characteristic<br>*Verified with time evolution of the maximum power point or with the photocurrent at maximum power point; see ref. 7 for details.*	☐ Yes ☒ No	*State where this information can be found in the text.* / MPP tracking is not necessary for organic solar cells.

3. Hysteresis or any other unusual behaviour

Item	Yes/No	Response
Description of the unusual behaviour observed during the characterization	☐ Yes ☒ No	*State where this information can be found in the text.* / No hysteresis or other unusual behavior was observed during the characterization of the devices.
Related experimental data	☐ Yes ☒ No	*State where this information can be found in the text.* / No hysteresis or other unusual behavior was observed during the characterization of the devices.

4. Efficiency

Item	Yes/No	Response
External quantum efficiency (EQE) or incident photons to current efficiency (IPCE)	☒ Yes ☐ No	The EQE data were measured by a Solar Cell Spectral Response Measurement System (RE-R, Enlitech). / *Explain why this information is not reported/not relevant.*
A comparison between the integrated response under the standard reference spectrum and the response measure under the simulator	☒ Yes ☐ No	The integrated Jcal values from EQE spectra are consistent with Jsc values from J-V measurements (Figure 5 and Table 1). / *Explain why this information is not reported/not relevant.*

November 2017

For tandem solar cells, the bias illumination and bias voltage used for each subcell

☐ Yes — *State where this information can be found in the text.*
☒ No — Only single-junction solar cells.

5. Calibration

Light source and reference cell or sensor used for the characterization

☒ Yes — Described in the section of "Methods" in the Manuscript.
☐ No — *Explain why this information is not reported/not relevant.*

Confirmation that the reference cell was calibrated and certified

☒ Yes — described in the section of "Methods" in the Manuscript.
☐ No — *Explain why this information is not reported/not relevant.*

Calculation of spectral mismatch between the reference cell and the devices under test

☒ Yes — The light spectrum used for measurements matches well with the reference silicon cell, and we did not calculate the spectral mismatch between the reference cell and the tested devices.
☐ No — *Explain why this information is not reported/not relevant.*

6. Mask/aperture

Size of the mask/aperture used during testing

☒ Yes — A mask with area of 0.05979 cm2 (certified by National Institute of Metrology, China) was used for test.
☐ No — *Explain why this information is not reported/not relevant.*

Variation of the measured short-circuit current density with the mask/aperture area

☐ Yes — *State where this information can be found in the text.*
☒ No — All the devices were tested with a mask.

7. Performance certification

Identity of the independent certification laboratory that confirmed the photovoltaic performance

☒ Yes — Device performance was certified by National Institute of Metrology, China.
☐ No — *Explain why this information is not reported/not relevant.*

A copy of any certificate(s)
Provide in Supplementary Information

☒ Yes — Supplementary Information, Certification Reports.
☐ No — *Explain why this information is not reported/not relevant.*

8. Statistics

Number of solar cells tested

☒ Yes — Number of solar cells tested is provided in Figure 5 and Table 1.
☐ No — *Explain why this information is not reported/not relevant.*

Statistical analysis of the device performance

☒ Yes — Statistical analysis is provided in Figure 5 and Table 1.
☐ No — *Explain why this information is not reported/not relevant.*

9. Long-term stability analysis

Type of analysis, bias conditions and environmental conditions
For instance: illumination type, temperature, atmosphere humidity, encapsulation method, preconditioning temperature

☐ Yes — *State where this information can be found in the text.*
☒ No — As we mainly focus on structure-properties relationship analysis, we have not done stability measurements on our devices.
